# Interferon-α stimulates DExH-box helicase 58 to prevent hepatocyte ferroptosis

**DOI:** 10.1186/s40779-024-00524-9

**Published:** 2024-04-15

**Authors:** Kai-Wei Jia, Ren-Qi Yao, Yi-Wen Fan, Ding-Ji Zhang, Ye Zhou, Min-Jun Wang, Li-Yuan Zhang, Yue Dong, Zhi-Xuan Li, Su-Yuan Wang, Mu Wang, Yun-Hui Li, Lu-Xin Zhang, Ting Lei, Liang-Chen Gui, Shan Lu, Ying-Yun Yang, Si-Xian Wang, Yi-Zhi Yu, Yong-Ming Yao, Jin Hou

**Affiliations:** 1 National Key Laboratory of Medical Immunology & Institute of Immunology, Naval Medical University, Shanghai, 200433 China; 2 Department of General Surgery, the First Medical Center of Chinese PLA General Hospital, Beijing, 100853 China; 3 Translational Medicine Research Center, Medical Innovation Research Division and Fourth Medical Center of the Chinese PLA General Hospital, Beijing, 100853 China; 4 Center for Immunotherapy, Chinese Academy of Medical Sciences, Beijing, 100005 China

**Keywords:** Ischemia/reperfusion (I/R), DExH-box helicase 58 (DHX58), Glutathione peroxidase 4 (GPX4), m^6^A modification, YT521-B homology domain containing 2 (YTHDC2)

## Abstract

**Background:**

Liver ischemia/reperfusion (I/R) injury is usually caused by hepatic inflow occlusion during liver surgery, and is frequently observed during war wounds and trauma. Hepatocyte ferroptosis plays a critical role in liver I/R injury, however, it remains unclear whether this process is controlled or regulated by members of the DEAD/DExH-box helicase (DDX/DHX) family.

**Methods:**

The expression of DDX/DHX family members during liver I/R injury was screened using transcriptome analysis. Hepatocyte-specific *Dhx58* knockout mice were constructed, and a partial liver I/R operation was performed. Single-cell RNA sequencing (scRNA-seq) in the liver post I/R suggested enhanced ferroptosis by *Dhx58*^*hep−/−*^. The mRNAs and proteins associated with DExH-box helicase 58 (DHX58) were screened using RNA immunoprecipitation-sequencing (RIP-seq) and IP-mass spectrometry (IP-MS).

**Results:**

Excessive production of reactive oxygen species (ROS) decreased the expression of the IFN-stimulated gene *Dhx58* in hepatocytes and promoted hepatic ferroptosis, while treatment using IFN-α increased DHX58 expression and prevented ferroptosis during liver I/R injury. Mechanistically, DHX58 with RNA-binding activity constitutively associates with the mRNA of glutathione peroxidase 4 (GPX4), a central ferroptosis suppressor, and recruits the m^6^A reader YT521-B homology domain containing 2 (YTHDC2) to promote the translation of *Gpx4* mRNA in an m^6^A-dependent manner, thus enhancing GPX4 protein levels and preventing hepatic ferroptosis.

**Conclusions:**

This study provides mechanistic evidence that IFN-α stimulates DHX58 to promote the translation of m^6^A-modified *Gpx4* mRNA, suggesting the potential clinical application of IFN-α in the prevention of hepatic ferroptosis during liver I/R injury.

**Supplementary Information:**

The online version contains supplementary material available at 10.1186/s40779-024-00524-9.

## Background

Ferroptosis is a form of regulated cell death characterized by iron-dependent lipid peroxidation to lethal levels [1–3]. It has been shown to play critical roles in a series of physiological and pathological processes, especially in ischemia/reperfusion (I/R) injury when excessive reactive oxygen species (ROS) are produced [4, 5]. The biochemical mechanism underlying ferroptosis is the accumulation of lethal ROS and iron Fenton reaction-induced lipid peroxides (LPOs) combined with the depletion of glutathione (GSH) and inactivation of the enzyme glutathione peroxidase 4 (GPX4), which is the central suppressor of ferroptosis by catalyzing the conversion of GSH to oxidized GSH (GSSG) and eliminating LPOs [6]. However, as liver I/R injury is the leading cause of surgery-related liver injury, it also commonly occurs during war wounds or trauma [7], the lack of effective and safe clinical precautionary or therapeutic measures is still the main problem in preventing hepatic ferroptosis [8], especially for the potential approach of enhancing GPX4 expression or activity.

DEAD/DExH-box helicase (DDX/DHX) members constitute the largest family of RNA helicases [9, 10]. These members confer RNA binding and unwinding properties and are critical for RNA metabolism, including RNA recognition, modification, splicing, transport, degradation, and translation [11, 12]. Some also belong to the interferon (IFN)-stimulated gene (ISG) family, whose expression can be induced by IFN treatment. Additionally, certain genes within this family have the ability to stimulate IFN production, such as *DDX58* [also known as retinoic acid-inducible gene-I (RIG-I)] [13, 14], and *DHX58* [also known as laboratory of genetics and physiology 2 (LGP2)] [15, 16]. DDX58 (RIG-I) serves as an intracellular sensor for pathogen-associated molecular patterns present in viral RNA. It possesses a DExD/H-box RNA helicase domain that exhibits ATP hydrolysis activity, along with a C-terminal repressor domain (RD) embedded within the C-terminal domain (CTD) [17]. Both the helicase and RD domains are necessary for recognizing dsRNA and 5’-triphosphate RNA in a synergistic manner [18]. Upon recognizing viral RNA, the two N-terminal tandem caspase-recruiting domains (CARDs) of DDX58 can activate downstream type I IFN production. Compared with DDX58, DHX58 (LGP2) lacks the N-terminal CARDs responsible for recruiting and interacting with downstream antiviral components. DHX58 is regarded as a non-canonical RNA-binding protein (RBP) due to its ability to associate with RNAs [19]. We previously focused on the roles of DDX/DHX family members, including DDX58 and DDX46, in regulating liver physiopathology and inflammation [20–22]. However, the potential roles of DDX/DHX family members in the development of hepatic ferroptosis remain unknown up to now.

The regulation of gene expression in eukaryotic cells relies heavily on mRNA metabolism, which is strictly controlled by post-transcriptional modifications, including the highly prevalent N^6^-methyladenosine (m^6^A) modification within RNA [23]. The installation of m^6^A modification is mediated by m^6^A “writers”, such as the methyltransferase complex methyltransferase-like 3 (METTL3) and METTL14, while their removal is facilitated by m^6^A “erasers”, including the fat mass and obesity-related gene (*FTO*) and alkB homolog 5 (ALKBH5). The fate and function of m^6^A-modified RNAs are primarily administered by m^6^A “readers”, such as members of the YT521-B homology (YTH) domain family (including YTHDF1, YTHDF2, YTHDF3, YTHDC1, and YTHDC2). These readers regulate mRNA splicing, stability, transport, and translation by recognizing the m^6^A sites within the mRNAs [24, 25]. Notably, YTHDC2 exerts control over both mRNA translation efficiency and stability in an m^6^A-dependent manner [26]. However, the specific mechanism by which these m^6^A enzymes modify or read their corresponding mRNAs remains unclear.

In this study, we screened the expression of DDX/DHX family members during liver I/R injury, and identified DHX58 as the most significantly downregulated member in ISG. Therefore, our focus was on elucidating the potential roles of hepatic DHX58 in the development of I/R injury, including its involvement in regulating hepatocyte death and RNA-binding activity. This study offers insights into understanding its underlying mechanism and provides a basis for preventing liver I/R injury.

## Methods

### Reagents

Antibodies against cyclooxygenase-2 (COX2; 12282), solute carrier family 7 member 11 (SLC7A11; 98051), V5-tag (13202), and horseradish peroxidase-conjugated secondary antibodies (7074 and 7076) were purchased from Cell Signaling Technology (Danvers, MA, USA). Antibodies specific to 4-hydroxynonenal (4-HNE; ab46545), malondialdehyde (MDA; ab243066), lymphocyte antigen 6 complex locus G (Ly6G; ab238132), F4/80 (ab300421), GPX4 (ab125066), METTL3 (ab195352), acyl-CoA synthetase long-chain family member 4 (ACSL4; ab155282), and YTHDC2 (ab220160) were purchased from Abcam (Cambridge, MA, USA). Antibodies specific to β-actin (A5441) and Flag-tag (F1804) were purchased from Sigma-Aldrich (St. Louis, MO, USA). The antibody specific to DHX58 (11355-1-AP) was purchased from Proteintech (Wuhan, China). Antibody specific to m^6^A (202003) was obtained from Synaptic Systems (Germany). Antibodies specific to CD45-Bv605 (63-0451-82) and CD11b-Percp.cy5.5 (45-0112-82) were obtained from Invitrogen (Carlsbad, CA, USA). Antibodies specific to F4/80-PE (123110) and Ly6G-FITC (127605) were purchased from BioLegend (San Diego, CA, USA). Protein G Agarose (P4691) and Anti-Flag M2 Affinity Gel (A2220) were from Sigma-Aldrich (St. Louis, MO, USA). Liproxstatin-1 (S7699), Z-VAD-FMK (Zvad; S7023), and necrostatin-1 (Nec-1; S8037) were purchased from SelleckChem (Houston, TX, USA). DMEM (11965092), fetal bovine serum (FBS; 10099141 C), and RPMI 1640 (11875093) were from Gibco (Shanghai, China).

### Animals

One hundred C57BL/6 mice were purchased from Joint Ventures Sipper BK Experimental Animal Co. (Shanghai, China). To generate hepatocyte-specific DHX58 deficiency, *Dhx58*^*f/f*^ mice (*n* = 4) were designed and constructed by ViewSolid Biotech (Beijing, China) using clustered regularly interspaced short palindromic repeats/CRISPR-associated 9 (CRISPR/Cas9) techniques, as previously described [27], and then hybridized with Alb-Cre transgenic mice (003574, *n* = 4) purchased from The Jackson Laboratory (Bar Harbor, ME, USA). Mouse genotyping was performed by PCR analysis of genomic DNA extracted from the tails, as previously described [28]. For the knockdown and overexpression of hepatic *Dhx58*, the adeno-associated virus serotype 8 (AAV8) constructs were established by OBiO Technology (Shanghai, China) as we previously described [21]. The shRNA target sequence of *Dhx58* was 5’-CCTGACTTGAAGCAACAATTT-3’. A single tail vein injection of 2 × 10^11^ AAV8 was administered, and the mice underwent I/R two weeks post injection. All animal experiments were performed in accordance with the National Institute of Health Guide for the Care and Use of Laboratory Animals, with the approval of the Scientific Investigation Board of Naval Medical University, Shanghai, China.

### Mouse models

For the liver I/R mouse model, male mice aged 8–10 weeks were anesthetized with pentobarbital sodium (100 mg/kg). After opening the abdominal cavity, an atraumatic vascular clamp was placed across the hepatic artery, portal vein, and bile duct to interrupt the left lateral and median lobes (70%) of the liver. After 60 min of inducing hepatic ischemia, the clamp was removed to initiate reperfusion. Sham mice underwent the same surgical treatment without vascular occlusion. The mice were sacrificed at the indicated reperfusion time points, and the serum and liver were collected immediately. To reduce ROS, N-acetylcysteine (NAC; A9165, Sigma, USA, 15 mg/kg in sterile phosphate buffered saline, intraperitoneal injection) or butylated hydroxyanisole (BHA; B1253, Sigma, USA, 50 mg/kg in corn oil, gavage) was administered twice daily for 2 d starting 48 h before liver I/R. To activate nuclear factor erythroid 2-related factor 2 (Nrf2) in vivo, 50 mg/kg dimethyl fumarate (DMF, s2586, SelleckChem, USA) was treated daily by gavage for 7 d before liver I/R. For the pharmacological inhibition of cell death, liproxstatin-1 (10 mg/kg), Zvad (10 mg/kg), or Nec-1 (1.65 mg/kg) were administered by intraperitoneal injection 30 min before surgery to inhibit ferroptosis, apoptosis, and necroptosis. Regarding the induction of hepatic ferroptosis, erastin (25 mg/kg) was injected intraperitoneally for 2 d at 12-h intervals, and ferric nitrilotriacetate (Fe-NTA; 22 mg/kg) was injected peritoneally for 3 h. The IFN-α treatment group was intraperitoneally injected with a single dose of IFN-α (1 × 10^6^ U/kg, 752804, BioLegend, USA) at 12 h before liver I/R.

### Single-cell RNA sequencing (scRNA-seq)

As described previously, mouse livers were digested to obtain single-cell suspensions A [20]. Dissociated single cells were stained with AO/PI for viability assessment using a Countstar Fluorescence Cell Analyzer. The scRNA-seq libraries were generated using the 10× Genomics Chromium Controller Instrument and Chromium Single Cell 3’V3.1 Reagent Kits (10× Genomics, Pleasanton, CA, USA) according to the recommendations of the manufacturer. scRNA-seq data analysis, including cell communication analysis, quantitative set analysis of gene expression (QuSAGE) analysis, and pathway analysis were performed by NovelBio Bio-Pharm Technology Co., Ltd., using the NovelBrain Cloud Analysis Platform as previous procedures [29–32]. To characterize the relative activation of a given gene set, such as pathway activation, we performed QuSAGE analysis, in which gene sets, including ferroptosis, described previously, were added. Pathway analysis was used to identify the significant pathways of the marker genes and differentially expressed genes according to the kyoto encyclopedia of genes and genomes (KEGG) database. Fisher’s exact test was used to select significant pathways, and the threshold of significance was defined by the *P*-value and false discovery rate (FDR).

### Isolation of primary hepatocytes and non-parenchymal cells (NPCs)

Primary hepatocytes and NPCs were isolated according to the protocol [33]. Briefly, the mouse liver was perfused in the inferior vena cava above the kidney with 25 ml perfusion buffer and then with 10 ml digestion buffer. Subsequently, the liver sack was ruptured with fine-tip forceps along the liver surface, and the cells were released using a cell lifter. The cells were filtered through a 70 μm cell strainer and then centrifuged at 50 *g* for 2 min. Only the hepatocytes were pelleted, whereas the other cells remained in the supernatant. Primary hepatocytes and NPCs were collected and purified for further experiments. The viability of primary hepatocytes was evaluated by trypan blue exclusion (> 90%).

### Human liver samples

Human normal liver tissues, obtained from distal normal liver tissues of liver hemangioma patients, and human liver tissues post I/R, obtained from biopsy approximately 3 h post reperfusion in donors during liver transplantation, were collected at Huashan Hospital. The donor livers for transplantation underwent 5 to 10 min of warm ischemia followed by 5 to 7 h of cold ischemia. The human subject study was approved by the human ethics committee of Huashan Hospital, Fudan University (KY2021-449).

### 4D label free proteomics analysis

The proteomics analysis was performed using PTM BIO (Hangzhou, China). Protein lysates from *Dhx58*^*f/f*^ and *Dhx58*^*hep−/−*^ livers were diluted and digested with trypsin. Tryptic peptides were dissolved in solvent A (0.1% formic acid and 2% acetonitrile/water) and directly loaded onto a home-made reversed-phase analytical column. Peptides were separated with a gradient from 6 to 24% solvent B (0.1% formic acid in acetonitrile) over 70 min, 24 to 35% in 14 min, climbing to 80% in 3 min, and then held at 80% for the last 3 min, all at a constant flow rate of 450 nl/min on a nanoElute UHPLC system (Bruker Daltonics, USA). The peptides were subjected to a capillary source, followed by the timsTOF Pro (Bruker Daltonics, USA) mass spectrometry (MS). Precursors and fragments were analyzed using a TOF detector with an MS/MS scan range of 100–1700 m/z. The resulting MS/MS data were processed using the MaxQuant search engine (v.1.6.15.0). Tandem mass spectra were searched against the human SwissProt database (20422 entries) and concatenated with a reverse-decoy database.

### Co-immunoprecipitation (Co-IP) and Western blotting

Cells and livers were collected and lysed using cell lysis buffer (9803, Cell Signaling Technology, MA, USA) supplemented with a protease inhibitor cocktail (539134, Calbiochem, Germany). The protein concentrations in the lysates were measured using a bicinchoninic acid (BCA) protein assay kit (23225, Pierce, USA) and equalized with cell lysis buffer. An equal amount of the extracts was used for immunoprecipitation, which was incubated with the antibodies and protein beads overnight, and the immune complex beads were washed and boiled with sample buffer, or the extracts were directly loaded and subjected to SDS-PAGE, transferred onto nitrocellulose membranes, and then blotted as we described previously [34]. β-actin was used as a loading control.

### RNA immunoprecipitation-sequencing (RIP-seq) and RIP-qRT-PCR

Primary hepatocytes seeded in a 15 cm dish at 85% confluence were cross-linked by ultraviolet light and harvested. RIP was performed using a Magna RIP™ RNA-Binding Protein Immunoprecipitation Kit (Millipore, USA) according to the instructions of the manufacturer. Cell lysates were subjected to RIP using rabbit IgG (Millipore, USA), which served as a negative control, or anti-DHX58 antibody (11355-1-AP, Proteintech, China). Input and co-immunoprecipitated RNAs were isolated using TRIzol reagent for sequencing and qRT-PCR.

### Study of m^6^A

m^6^A RIP was performed according to the instructions of the Magna RIP™ RNA-Binding Protein Immunoprecipitation Kit. Cell lysates were subjected to RIP using rabbit IgG (Millipore, USA), a negative control, or anti-m^6^A (202003, Synaptic Systems, Germany). Input and co-immunoprecipitated RNAs were isolated using TRIzol reagent for sequencing and qRT-PCR. Global m^6^A mRNA levels were quantified using an m^6^A RNA methylation assay kit (ab185912, Abcam, USA) following the protocol by the manufacturer. MeRIP-seq was performed by the Biotechnology Corporation (Shanghai, China).

### Polysome profiling

The procedures were performed according to a previously described protocol [35]. The hepatocytes were collected with polysome extraction buffer [20 mmol/L Tris-HCl pH 7.5, 100 mmol/L KCl, 5 mmol/L MgCl_2_, 0.5% Nonidet P-40, protease inhibitors, RNase inhibitors, and 100 µg/ml cycloheximide (CHX)], and the sample was loaded onto a 10–50% w/v sucrose gradient. The gradients were centrifuged at 4 ℃ for 90 min in a SW41Ti swinging bucket rotor at 39,000 rpm. The gradients were fractioned using a Gradient Station (BioComp, Canada) equipped with an ECONO UV monitor (BioRad, USA) and collected using a fraction collector (FC203B, Gilson, USA). Total RNA from the indicated fractions was extracted using TRIzol reagent for qRT-PCR analysis. For more details, please see Additional file 1: Materials and methods.

### Statistical analysis

Data are presented as the mean ± SD of one representative of at least three independent experiments. Statistical comparisons between experimental groups were analyzed by the Student’s *t*-test in SPSS 17.0 (Chicago, IL, USA), and a two-tailed *P* < 0.05 was taken to indicate statistical significance.

## Results

### Excessive ROS during liver I/R injury decreases hepatic DHX58 expression

**Fig. 1:**
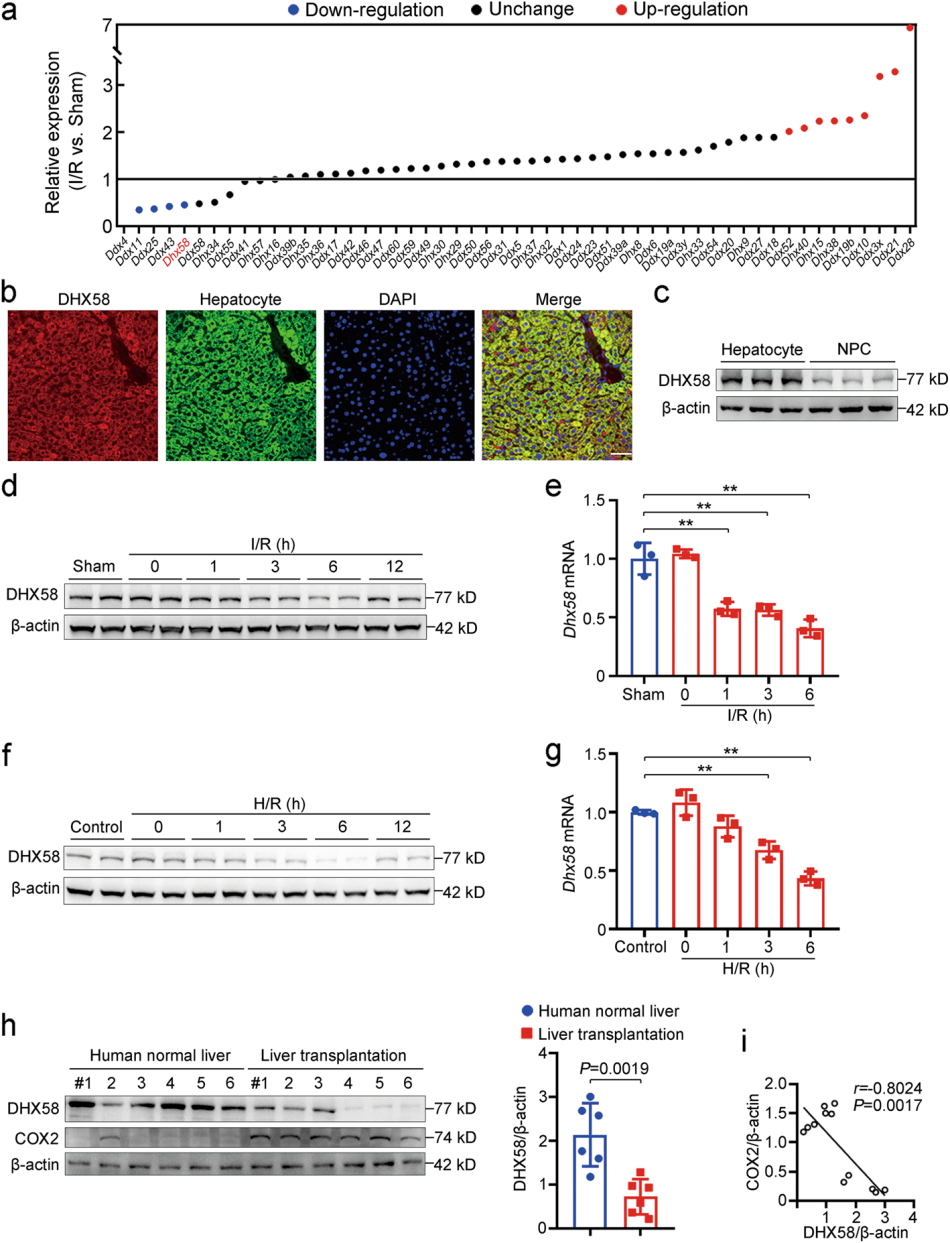
Hepatic DHX58 expression is markedly decreased during liver I/R injury. **a** Expression of hepatic DDX/DHX family members following liver I/R injury. **b** Immunofluorescence staining images of DHX58, hepatocyte and DAPI in liver sections analyzed by confocal microscopy. Scale bar = 50 µm. **c** DHX58 protein level in the isolated hepatocytes and non-parenchymal cells (NPCs) of the liver was examined by Western blotting. **d** DHX58 protein level in liver tissues after I/R injury was examined by Western blotting. **e** *Dhx58* mRNA level in liver tissues after I/R injury was examined by qRT-PCR. **f** DHX58 protein level in primary hepatocytes following H/R injury was examined by Western blotting. **g** *Dhx58* mRNA level in primary hepatocytes following H/R injury was examined by qRT-PCR. **h** DHX58 and COX2 in human normal liver tissues, obtained from distal normal liver tissues of liver hemangioma patients, and human liver tissues post I/R, obtained from biopsy approximately 3 h post reperfusion in donors during liver transplantation, were examined by Western blotting. The donor livers for transplantation underwent 5 to 10 min of warm ischemia followed by 5 to 7 h of cold ischemia. Quantified DHX58 expression was shown. **i** The correlation between DHX58 and COX2 in (**h**) was analyzed by Pearson’s correlation coefficient assay. Data are shown as mean ± SD (*n* = 3 or indicated) or photographs from one representative of three independent experiments. ^**^P < 0.01 compared with the sham/control group by Student’s two-tailed *t*-test. DHX58 DExH-box helicase 58, I/R ischemia/reperfusion, DAPI 4’,6-diamidino-2-phenylindole, H/R hypoxia/re-oxygenation, COX2 cyclooxygenase-2, SD standard deviation

To comprehensively elucidate the potential roles of DDX/DHX family members in the development of liver I/R injury, we conducted an analysis on their expression and found that *Dhx58* was one of the five family members (*Ddx4*, *Ddx11*, *Ddx25*, *Ddx43*, and *Dhx58*) with the most markedly decrease in expression after I/R in the liver (Fig. 1a; Additional file 1: Fig. S1a). The basal expression of the first 4 genes was too low to be analyzed (Additional file 1: Fig.S1b), thus our focus shifted to *Dhx58*. DHX58 expression in the liver was mainly located in parenchymal hepatocytes (Fig. 1b, c). Both protein and mRNA levels of *DHX58* were confirmed to be reduced within 6 h in the liver following I/R in vivo (*P* < 0.01, Fig. 1d, e) and in primary hepatocytes after H/R in vitro (*P* < 0.01, Fig. 1f, g). Moreover, we collected human liver samples from donors after reperfusion during transplantation and observed a significant decrease in hepatic DHX58 expression (*P* = 0.0019) during I/R injury, accompanied by an increase of liver COX2 expression. Furthermore, a negative correlation was found between the levels of DHX58 and COX2 (*r* = -0.8024, *P* = 0.0017) (Fig. 1h, i). Therefore, the expression of DHX58 in hepatocytes is decreased in response to liver I/R injury.

**Fig. 2:**
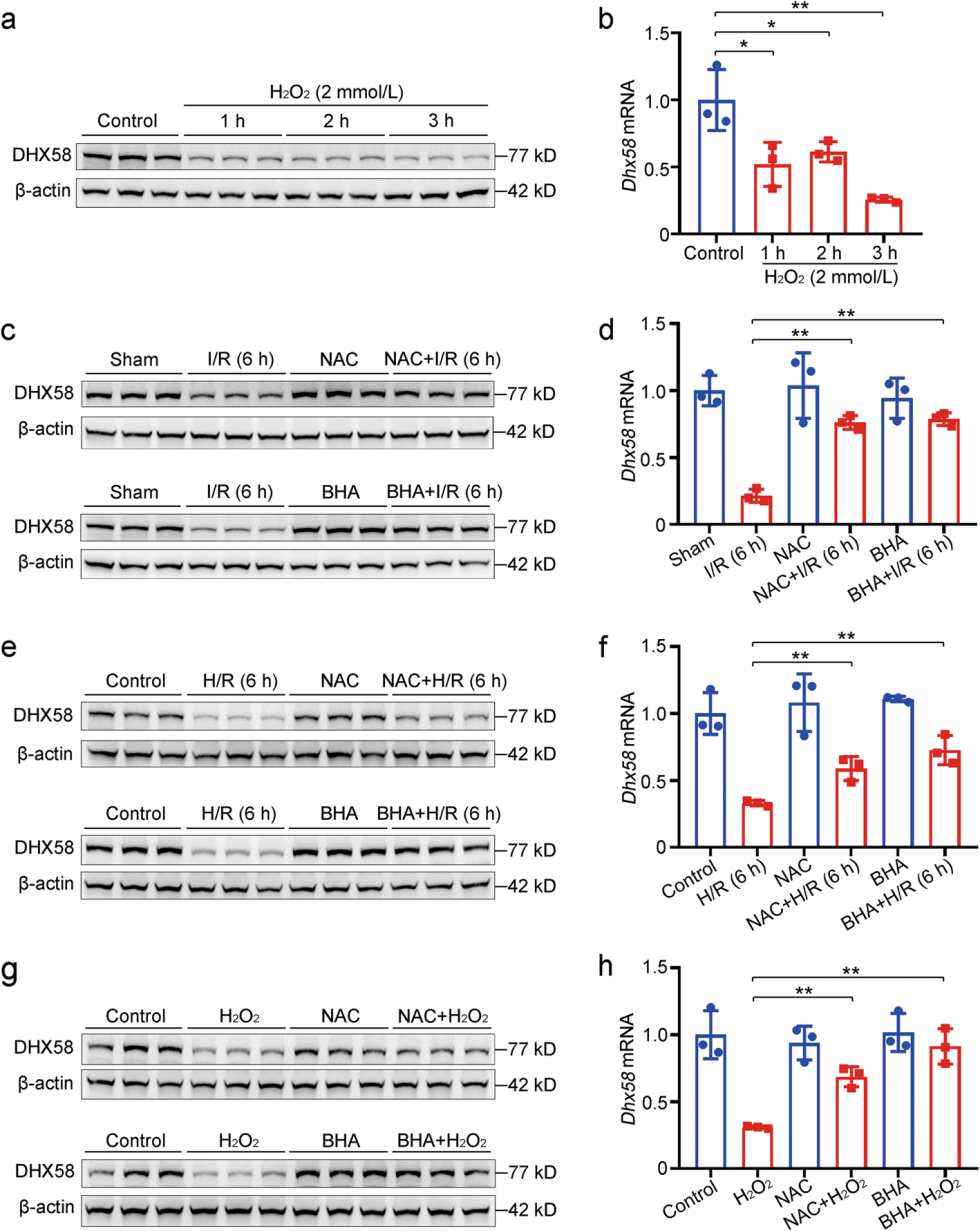
Excessive ROS production during liver I/R injury decreases DHX58 expression. **a** DHX58 protein level in primary hepatocytes treated with H_2_O_2_ was examined by Western blotting. **b** *Dhx58* mRNA level in primary hepatocytes treated with H_2_O_2_ was examined by qRT-PCR. **c** DHX58 protein level in liver tissues with NAC or BHA pre-treatment and then I/R was examined by Western blotting. **d** *Dhx58* mRNA level in liver tissues with NAC or BHA pre-treatment and then I/R was examined by qRT-PCR. **e** DHX58 protein level in primary hepatocytes with NAC or BHA pre-treatment and then H/R was examined by Western blotting. **f** *Dhx58* mRNA level in primary hepatocytes with NAC or BHA pre-treatment and then H/R was examined by qRT-PCR. **g** DHX58 protein level in primary hepatocytes with NAC or BHA pre-treatment and then H_2_O_2_ (2 mmol/L) administration for 3 h was examined by Western blotting. **h** *Dhx58* mRNA level in primary hepatocytes with NAC or BHA pre-treatment and then H_2_O_2_ (2 mmol/L) administration for 3 h was examined by qRT-PCR. Data are shown as mean ± SD (*n* = 3) or photographs from one representative of three independent experiments. ^*^P < 0.05, ^**^P < 0.01. ROS reactive oxygen species, I/R ischemia/reperfusion, DHX58 DExH-box helicase 58, NAC N-acetylcysteine, BHA butylated hydroxyanisole, H/R hypoxia/re-oxygenation, SD standard deviation

The excessive production of ROS is critical for initiating I/R injury [36]. Therefore, we found that H_2_O_2_ was responsible for the decrease in DHX58 levels in primary hepatocytes (*P* < 0.05 or *P* < 0.01, Fig. 2a, b), as well as in the human hepatocyte HHL5 cell line (*P* < 0.05 or *P* < 0.01, Additional file 1: Fig. S2a, b). The expression of DHX58 was also decreased by another ROS-generating compound, menadione (*P* < 0.01, Additional file 1: Fig. S2c, d). Using NAC or BHA to scavenge ROS, the decreased DHX58 levels were alleviated in the liver after I/R treatment in vivo (*P* < 0.01, Fig. 2c, d) and in primary hepatocytes treated with H/R or H_2_O_2_ in vitro (*P* < 0.01, Fig. 2e-h). Moreover, these treatments resulted in decreased serum ALT and AST levels (*P* < 0.05 or *P* < 0.01), indicating a reduction in liver damage (Additional file 1: Fig. S2e). Additionally, DMF-induced activation of the antioxidant Nrf2 mitigated the reduction of DHX58 in the liver after I/R and in primary hepatocytes following H/R (*P* < 0.01, Additional file 1: Fig. S2f-i). Taken together, excessive ROS production during I/R injury leads to a reduction in hepatic DHX58 expression, which may contribute to the development of liver I/R injury.

### Decreased DHX58 promotes hepatic ferroptosis and I/R injury

**Fig. 3:**
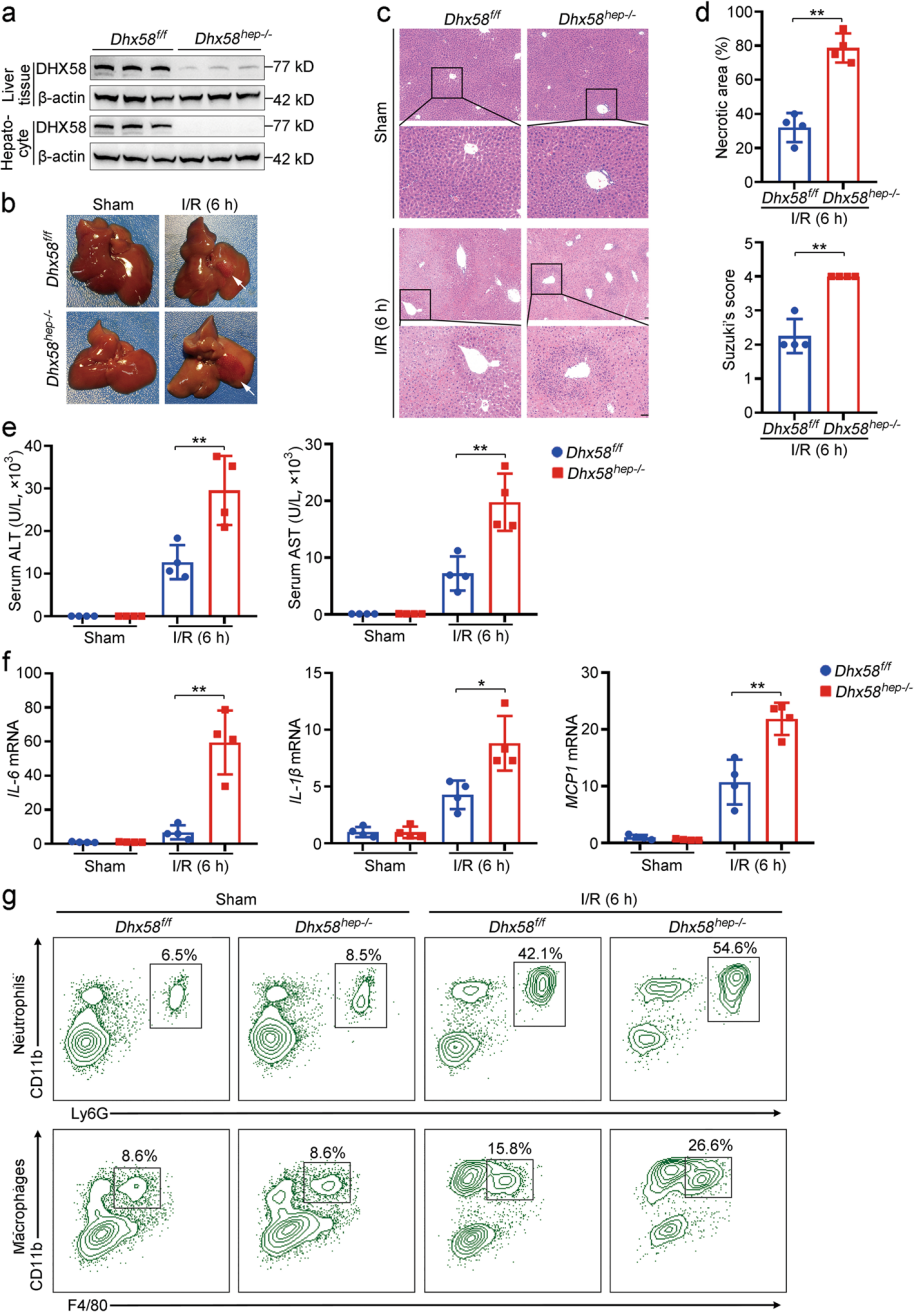
Decreased DHX58 aggravates liver I/R injury. **a** DHX58 protein level in liver tissues and isolated hepatocytes from *Dhx58*^*f/f*^ and *Dhx58*^*hep−/−*^ mice was confirmed by Western blotting. Liver I/R injury was administrated in *Dhx58*^*f/f*^ and *Dhx58*^*hep/−*^ mice, liver injury was shown by gross appearances of representative livers, and white arrow indicated injured area (**b**), liver pathology was analyzed by HE staining (**c**), necrotic area and Suzuki’s score were analyzed (*n* = 4) (**d**), serum ALT and AST were examined (*n* = 4) (**e**), *IL-6*, *IL-1β*, and *MCP1* mRNA levels in liver tissues were examined by qRT-PCR (*n* = 4) (**f**), infiltration of neutrophils and macrophages were analyzed by flow cytometry (**g**). Scale bar = 20 μm. Data are shown as mean ± SD or photographs from one representative of three independent experiments. ^*^P < 0.05, ^**^P < 0.01. DHX58 DExH-box helicase 58, I/R ischemia/reperfusion, HE hematoxylin-eosin, ALT alanine aminotransferase, AST aspartate aminotransferase, IL-6 interleukin-6, IL-1β interleukin-1β, MCP1 monocyte chemoattractant protein 1

To investigate the potential role of decreased DHX58 levels in developing liver I/R injury, we constructed hepatocyte-specific *Dhx58* knockout mice *Dhx58*^*hep−/−*^ (Fig. 3a; Additional file 1: Fig. S3a, b). The *Dhx58*^*hep−/−*^ livers seemed normal but exhibited significantly more severe liver damage (*P* < 0.01, Fig. 3b-d), higher serum ALT and AST (*P* < 0.01, Fig. 3e), elevated mRNAs of inflammatory cytokines [interleukin-6 (IL-6), IL-1β, monocyte chemoattractant protein 1 (MCP1)] (*P* < 0.05 or *P* < 0.01, Fig. 3f), and enhanced infiltration of inflammatory cells (neutrophils and macrophages; Fig. 3g; Additional file 1: Fig. S3c, d) during liver I/R injury compared with those in *Dhx58*^*f/f*^ livers. Moreover, primary hepatocytes isolated from *Dhx58*^*hep−/−*^ mice showed lower cell viability when subjected to H/R in vitro (*P* < 0.01), whereas DHX58 overexpression in primary hepatocytes improved cell viability under H/R compared to their respective controls (*P* < 0.01) (Additional file 1: Fig. S3e). Thus, the decreased expression of DHX58 during I/R may potentially contribute to liver injury.

**Fig. 4:**
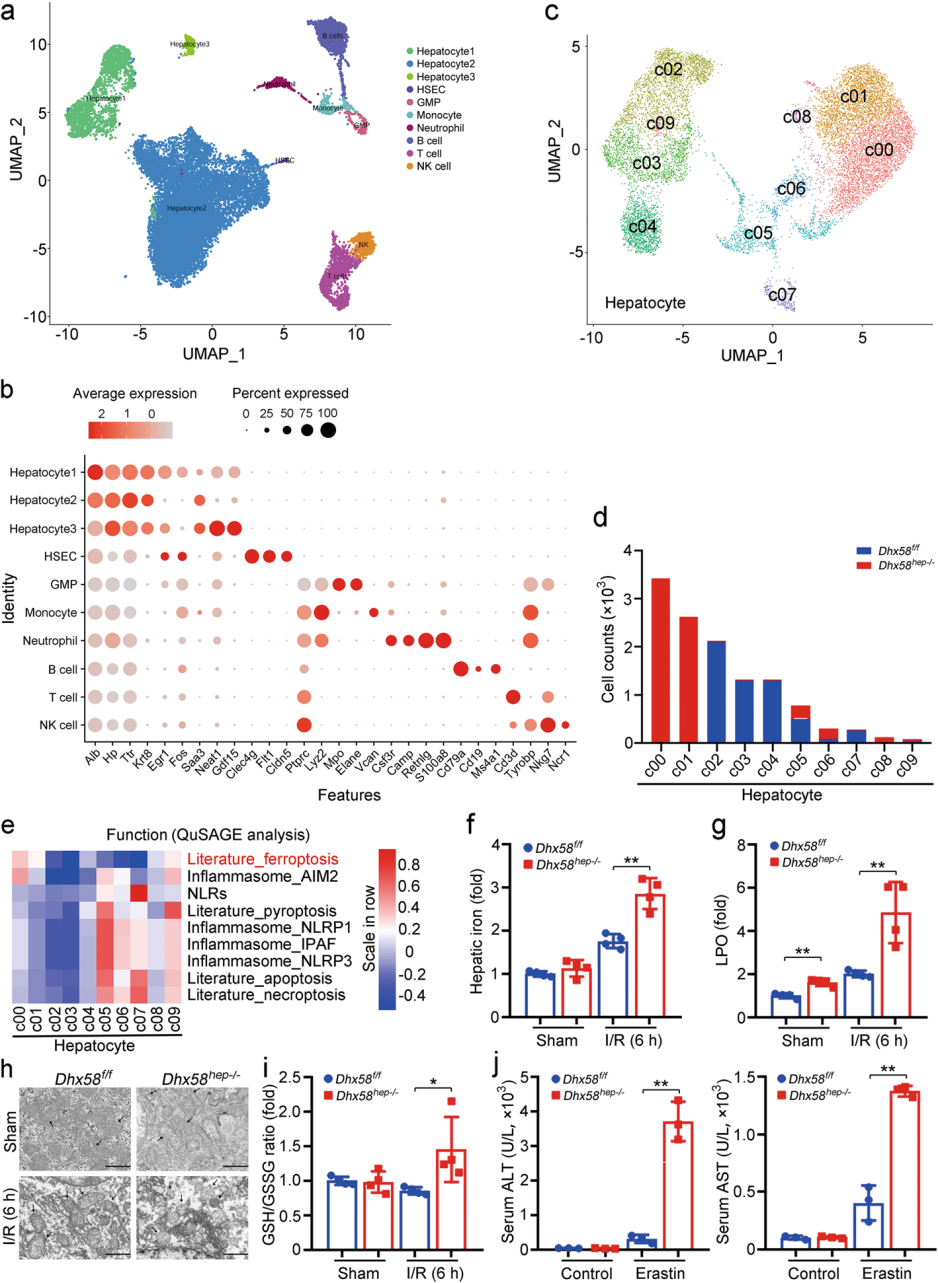
The downregulation of DHX58 expression following I/R injury promotes ferroptosis in hepatocyte. **a** UMAP visualization of cells in the livers of *Dhx58*^*f/f*^ and *Dhx58*^*hep−/−*^ mice underwent sham or I/R, and each dot corresponded to one single cell colored according to the cell cluster. **b** The bubble diagram of signature genes for each cell type is displayed. UMAP visualization of hepatocytes in I/R-treated *Dhx58*^*f/f*^ and *Dhx58*^*hep−/−*^ mice was shown as indicated (**c**), cell counts of *Dhx58*^*f/f*^ and *Dhx58*^*hep−/−*^ mice in each hepatocyte cluster were shown (**d**), and functional gene enrichment in each hepatocyte cluster by QuSAGE analysis was shown (**e**). Liver I/R injury was administrated in *Dhx58*^*f/f*^ and *Dhx58*^*hep/−*^ mice, hepatic iron (**f**) and LPO (**g**) levels were analyzed accordingly (*n* = 4), representative transmission electron microscope images show the morphology of ferroptosis in hepatocyte with black arrows indicated mitochondrion and lipid droplets (**h**), GSH and GSSG were analyzed and the ratio was calculated (*n* = 4) (**i**). Scale bar = 20 μm (**f**) and 2 μm (**h**). **j** Serum ALT and AST of *Dhx58*^*f/f*^ and *Dhx58*^*hep−/−*^ mice treated with ferroptosis inducer erastin (*n* = 3). Data are shown as mean ± SD or photographs from one representative of three independent experiments. ^*^P < 0.05, ^**^P < 0.01. DHX58 DExH-box helicase 58, I/R ischemia/reperfusion, UMAP uniform manifold approximation and projection, QuSAGE quantitative set analysis of gene expression, LPO lipid peroxide, GSH glutathione, GSSG oxidized glutathione, ALT alanine aminotransferase, AST aspartate aminotransferase, SD standard deviation, HSEC hepatic sinusoid endothelium cell, GMP granulocyte-monocyte progenitor, NK natural killer, AIM2 absent in melanoma 2, NLRs nucleotide-binding leucine-rich repeat receptors, NLRP1 nucleotide-binding oligomerization domain-like receptor protein 1, IPAF interleukin-1β-converting enzyme-protease activating factor

To clarify the underlying mechanism of *Dhx58*^*hep−/−*^-induced liver I/R injury, we performed scRNA-seq on the livers of *Dhx58*^*f/f*^ and *Dhx58*^*hep−/−*^ mice treated with sham or I/R. Intrahepatic cells were divided into 10 clusters, with the largest number of hepatocytes (Fig. 4a, b). Additionally, the scRNA-seq data of *Dhx58*^*f/f*^ and *Dhx58*^*hep−/−*^ livers after I/R were analyzed, and the cells were clustered into 17 populations (Additional file 1: Fig. S4a), with QuSAGE analysis showing ferroptosis enrichment in some of these cell clusters post I/R, including hepatocyte_c01, hepatocyte_c06, hepatocyte_c09, and hepatocyte_c13 (Additional file 1: Fig. S4b). Therefore, we re-clustered the hepatocytes of *Dhx58*^*f/f*^ and *Dhx58*^*hep−/−*^ livers under I/R, and divided them into 10 populations (Fig. 4c). QuSAGE analysis was specifically focused on the cell death pathways. The hepatocyte_c00 and c01, which exhibited significant enhancement in *Dhx58*^*hep−/−*^ livers (Fig. 4d), showed enrichment for ferroptosis as indicated by QuSAGE analysis (Fig. 4e). Moreover, pathway analysis revealed that ferroptosis was significantly enriched in hepatocyte_c00 and c01 (Additional file 1: Fig. S4c). Furthermore, cell phone analysis suggested potential interaction between hepatocyte_c00, c01, and other clusters, including neutrophils and monocytes (Additional file 1: Fig. S4d, e). Hence, the scRNA-seq data suggest that *Dhx58*^*hep−/−*^ may enhance ferroptosis in hepatocyte during I/R injury.

To confirm the promotion of ferroptosis in hepatocytes by *Dhx58*^*hep−/−*^, livers from *Dhx58*^*hep−/−*^ mice were examined after I/R injury. The results revealed an increase in ROS levels (Additional file 1: Fig. S5a), heightened 4-HNE levels (Additional file 1: Fig. S5b, c), elevated hepatic iron accumulation (*P* < 0.01, Fig. 4f), strengthened lipid peroxidation (*P* < 0.01, Fig. 4g), raised MDA levels (Additional file 1: Fig. S5d, e), augmented mitochondrial damage (Fig. 4h), increased TUNEL staining intensity (Additional file 1: Fig. S5f), and enhanced GSH/GSSG ratio (*P* < 0.05, Fig. 4i) compared to those in *Dhx58*^*f/f*^ livers. In addition, using the respective inhibitors of ferroptosis, apoptosis, or necroptosis in vivo, only inhibition of ferroptosis abolished the differences in liver I/R injury between *Dhx58*^*f/f*^ and *Dhx58*^*hep−/−*^ mice (Additional file 1: Fig. S5g-j), confirming *Dhx58*^*hep−/−*^ promotes ferroptosis in hepatocyte. We also knocked down or overexpressed hepatic *Dhx58* through AAV8-mediated gene delivery. The levels of serum ALT (*P* < 0.01) and AST (*P* < 0.05), hepatic iron (*P* < 0.05), LPO (*P* < 0.05), and the GSH/GSSG ratio (*P* < 0.01) were significantly altered in response to *Dhx58* knockdown, promoting I/R-induced liver injury and ferroptosis in hepatocyte, whereas DHX58 overexpression alleviated I/R-induced liver injury and ferroptosis in hepatocyte [serum ALT (*P* < 0.01) and AST (*P* < 0.05 or *P* < 0.01), hepatic iron (*P* < 0.05), LPO (*P* < 0.05), GSH/GSSG ratio (*P* < 0.01)] (Additional file 1: Fig. S6a-h). Furthermore, serum ALT and AST (*P* < 0.01), hepatic iron (*P* < 0.01), LPO (control: *P* < 0.05, erastin: *P* < 0.01), and the GSH/GSSG ratio (*P* < 0.01) were enhanced by *Dhx58*^*hep−/−*^ following ferroptosis inducer erastin treatment (Fig. 4j; Additional file 1: Fig. S6i-k). Following another ferroptosis inducer Fe-NTA treatment, serum ALT and AST (*P* < 0.01), hepatic iron (*P* < 0.05), LPO (control: *P* < 0.01, Fe-NTA: *P* < 0.05), and the GSH/GSSG ratio (*P* < 0.01) were also enhanced by *Dhx58*^*hep−/−*^ (Additional file 1: Fig. S6l-o). Moreover, cell viability was inhibited by DHX58 deficiency (*P* < 0.01) while enhanced by DHX58 overexpression (*P* < 0.05) in primary hepatocytes in vitro (Additional file 1: Fig. S6p, q). Taken together, our findings suggest that downregulation of DHX58 expression contributes to the induction of ferroptosis in hepatocyte during liver I/R injury.

### DHX58 associates and promotes the translation of *Gpx4* mRNA

**Fig. 5:**
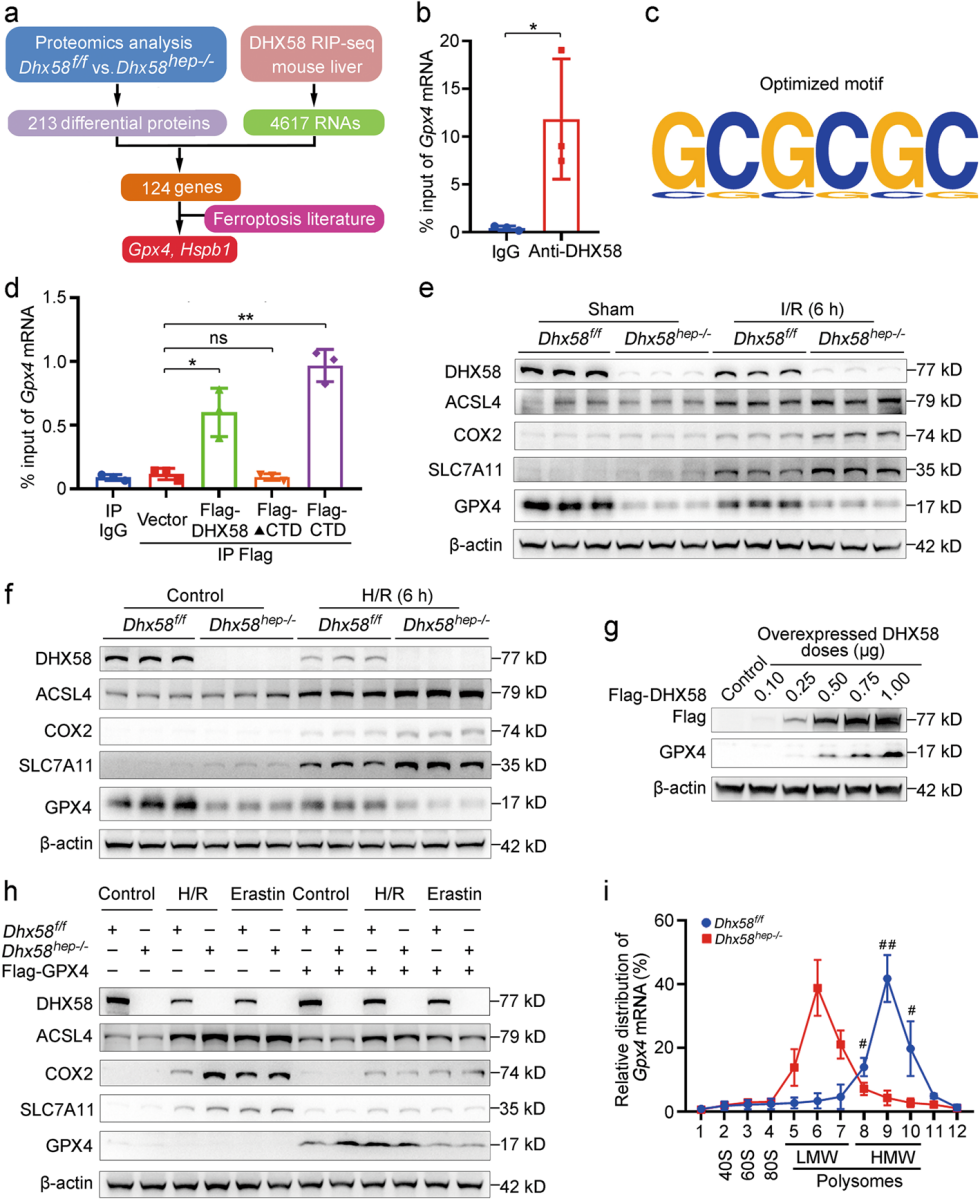
DHX58 associates *Gpx4* mRNA and promotes its translation. **a** Schematic workflow of DHX58 downstream targets analysis. **b** The association between DHX58 and *Gpx4* mRNA in primary hepatocytes were determined by RIP-qRT-PCR. ^*^P < 0.05. **c** The top motif identified by HOMER of DHX58-bound peaks in *Gpx4* mRNA. **d** The association between the CTD domain of DHX58 and endogenous *Gpx4* mRNA in primary hepatocytes was determined by RIP-qRT-PCR. ^*^P < 0.05, ^**^P < 0.01, ns non-significant. **e** DHX58, ACSL4, COX2, SLC7A11, and GPX4 protein levels in liver tissues of *Dhx58*^*f/f*^ and *Dhx58*^*hep−/−*^ mice after I/R injury were examined by Western blotting. **f** DHX58, ACSL4, COX2, SLC7A11, and GPX4 protein levels in primary hepatocytes from *Dhx58*^*f/f*^ and *Dhx58*^*hep−/−*^ mice following H/R injury were examined by Western blotting. **g** GPX4 protein level in DHX58-overexpressed primary hepatocytes was examined by Western blotting. **h** DHX58, ACSL4, COX2, SLC7A11, and GPX4 protein levels in primary hepatocytes from *Dhx58*^*f/f*^ or *Dhx58*^*hep−/−*^ mice with GPX4 overexpression and treatment of H/R or erastin were examined by Western blotting. **i** In primary hepatocytes from *Dhx58*^*f/f*^ and *Dhx58*^*hep−/−*^ mice, relative *Gpx4* mRNA distribution in each ribosome fractions was analyzed by qRT-PCR. Data are shown as mean ± SD (*n* = 3) or photographs from one representative of three independent experiments. ^#^*P* < 0.05, ^##^*P* < 0.01 vs. *Dhx58*^*f/f*^. ▲CTD C-terminal domain deleted, DHX58 DExH-box helicase 58, GPX4 glutathione peroxidase 4, RIP RNA immunoprecipitation, HOMER hypergeometric optimization of motif enrichment, CTD C-terminal domain, ACSL4 acyl-CoA synthetase long chain family member 4, COX2 cyclooxygenase-2, SLC7A11 solute carrier family 7 member 11, I/R ischemia/reperfusion, H/R hypoxia/re-oxygenation, LMW low molecular weight, HMW high molecular weight, SD standard deviation

The mechanism by which DHX58 inhibits ferroptosis was investigated. The proteomics screening of *Dhx58*^*f/f*^ and *Dhx58*^*hep−/−*^ livers revealed a total of 213 differentially expressed proteins. DHX58, belonging to the DDX/DHX family with RNA-binding activity, was subjected to RIP-seq using the DHX58 antibody, resulting in the identification of 4617 RNAs. The intersection of these two sets was 124 genes, and together with the literature search of ferroptosis, *Gpx4* and *Hspb1* were suggested to be differentially expressed between *Dhx58*^*f/f*^ and *Dhx58*^*hep−/−*^ livers, with their mRNAs levels being associated with DHX58 (Fig. 5a). Using RIP-qRT-PCR, we found that only *Gpx4* mRNA was stably associated with DHX58 (*P* < 0.05, Fig. 5b), with a sequence motif within *Gpx4* mRNA enriched in the RIP-seq peaks (Fig. 5c). It was determined that the CTD of DHX58 is responsible for this specific interaction. The binding capacity of the CTD to *Gpx4* mRNA closely resembles that of full-length DHX58 (*P* < 0.01, Fig. 5d). The presented data provide evidence for the association between DHX58 and *Gpx4* mRNA.

The ferroptosis suppressor *Gpx4* was then examined in the liver in vivo and in primary hepatocytes in vitro. *Dhx58*^*hep−/−*^ resulted in a reduction of GPX4 protein levels, while its mRNA level did not show significant change (Fig. 5e, f; Additional file 1: Fig. S7a, b). For other ferroptosis markers ACSL4, COX2, and SLC7A11, both protein and mRNA levels were induced by I/R in vivo and H/R in vitro, and their expression was further increased by *Dhx58*^*hep−/−*^ (Fig. 5e, f; Additional file 1: Fig. S7a, b), which is in accordance with the promoted ferroptosis in *Dhx58*^*hep−/−*^ liver. The DHX58-mediated increase in GPX4 and decrease in ACSL4, COX2, and SLC7A11 were also validated by knockdown or overexpression of *Dhx58*, both in the liver in vivo and in primary hepatocytes in vitro (Additional file 1: Fig. S7c-f). DHX58 overexpression in hepatocytes increased the protein level of GPX4 in a dose-dependent manner (Fig. 5g), confirming the binding of DHX58 to *Gpx4* mRNA to increase its protein level. Furthermore, in GPX4 overexpressed hepatocytes, the induced ferroptosis markers upon H/R or erastin administration were suppressed, and the differences between *Dhx58*^*f/f*^ and *Dhx58*^*hep−/−*^ were abolished (Fig. 5h), suggesting that *Dhx58*^*hep−/−*^ failed to promote ferroptosis under GPX4 overexpression, and the promotion of ferroptosis by *Dhx58*^*hep−/−*^ is dependent on GPX4 reduction. Thus, DHX58 can inhibit liver ferroptosis by binding to *Gpx4* mRNA and increasing its protein level.

To elucidate how DHX58 increases GPX4 protein levels, we first tested the degradation of GPX4 protein. Primary hepatocytes were treated with proteasome inhibitor MG132 and lysosome inhibitor chloroquine (CQ) to assess GPX4 expression. However, neither proteasome inhibitor nor lysosome inhibitor resulted in increased expression of GPX4 in *Dhx58*^*hep−/−*^ hepatocytes (Additional file 1: Fig. S7g), and these inhibitors also failed to alleviate differences in GPX4 protein levels mediated by DHX58 overexpression (Additional file 1: Fig. S7h), so we excluded that the proteasomal and lysosomal protein degradation of GPX4 was suppressed by DHX58. Next, *Gpx4* mRNA levels were not affected by DHX58 overexpression (Additional file 1: Fig. S7i), and its half-life was also unchanged by *Dhx58*^*hep−/−*^ (Additional file 1: Fig. S7j), suggesting that the stability of *Gpx4* mRNA was not influenced by DHX58. We then performed ribosome profiling of *Gpx4* mRNA, and found that its translation was suppressed by *Dhx58*^*hep−/−*^ (*P* < 0.05) (Fig. 5i). Taken together, we conclude that DHX58 associates with *Gpx4* mRNA and promotes its translation, thereby increasing GPX4 protein levels and preventing hepatic ferroptosis.

### DHX58 recruits YTHDC2 to enhance m^6^A-dependent translation of *Gpx4* mRNA

**Fig. 6:**
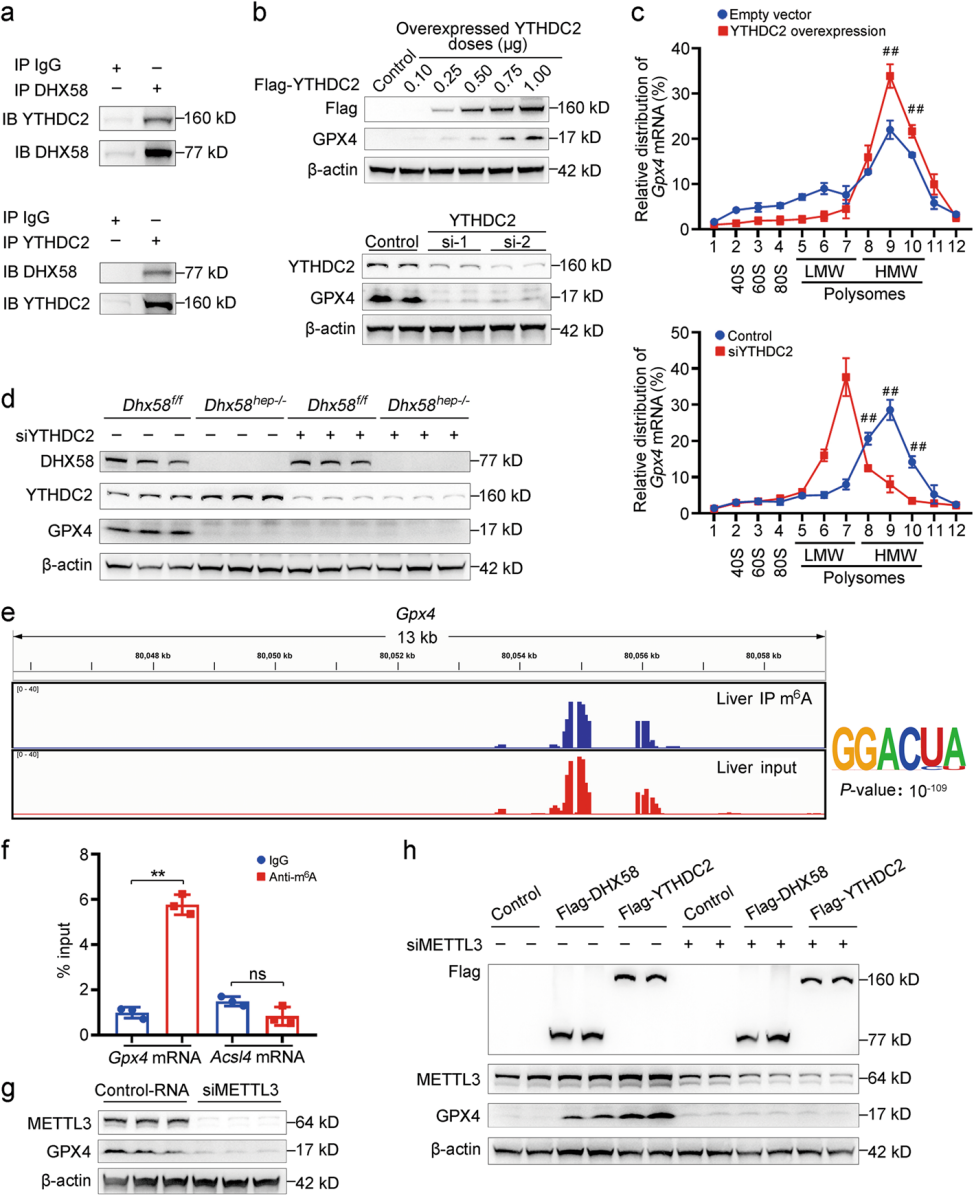
DHX58 recruits YTHDC2 to promote the translation of m^6^A-modified *Gpx4* mRNA. **a** The endogenous association between DHX58 and YTHDC2 in liver tissues was examined by Co-IP. **b** GPX4 protein level in primary hepatocytes with *Ythdc2* overexpression or knockdown was examined by Western blotting. **c** In primary hepatocytes with *Ythdc2* overexpression or knockdown, the relative distribution of *Gpx4* mRNA in each ribosome fraction was analyzed by qRT-PCR. ^##^*P* < 0.01 vs. Empty vector. **d** In primary hepatocytes from *Dhx58*^*f/f*^ and *Dhx58*^*hep−/−*^ mice with knockdown of *Ythdc2*, DHX58, YTHDC2, and GPX4 protein levels were examined by Western blotting. **e** Sequencing read clusters from MeRIP-seq analysis of *Gpx4* mRNA in primary hepatocytes and top consensus motif identified by HOMER with MeRIP-seq peaks. **f** m^6^A modification of *Gpx4* and *Acsl4* mRNAs in primary hepatocytes were examined by RIP-qRT-PCR. **g** GPX4 protein level in primary hepatocytes with knockdown of *Mettl3* was examined by Western blotting. **h** In primary hepatocytes transfected with Flag-tagged DHX58 or YTHDC2, together with *Mettl3* knockdown, Flag-tag, METTL3, and GPX4 protein levels were examined by Western blotting. Data are shown as mean ± SD (*n* = 3) or photographs from one representative of three independent experiments. ^**^P < 0.01. ns non-significant, Si-1 No.1 siRNA targeting YTHDC2, Si-2 No.2 siRNA targeting YTHDC2, DHX58 DExH-box helicase 58, YTHDC2 YT521-B homology domain containing 2, m^6^A N^6^-methyladenosine, Gpx4 glutathione peroxidase 4, Co-IP co-immunoprecipitation, MeRIP-seq methylated RNA immunoprecipitation sequencing, HOMER hypergeometric optimization of motif enrichment, Acsl4 acyl-CoA synthetase long chain family member 4, RIP RNA immunoprecipitation, METTL3 methyltransferase complex methyltransferase-like 3, CTD C-terminal domain, SD standard deviation, LMW low molecular weight, HMW high molecular weight

To elucidate the mechanism underlying DHX58-mediated promotion of *Gpx4* mRNA translation, we performed immunoprecipitation of Flag-tagged DHX58 from the lysates of transfected hepatocytes, employed MS to identify proteins associated with DHX58, and selected YTHDC2, an m^6^A reader with the highest protein score, as a potential candidate (Additional file 1: Fig. S8a-c). The constitutive endogenous association between DHX58 and YTHDC2 was validated (Fig. 6a), with the CTD domain of DHX58 and the R3H domain of YTHDC2 identified as responsible for their interaction (Additional file 1: Fig. S8d, e). Moreover, the expression of GPX4 protein was enhanced by YTHDC2 overexpression, similar to DHX58. Conversely, *Ythdc2* knockdown inhibited the GPX4 protein expression (Fig. 6b). However, the mRNA levels remained unchanged (Additional file 1: Fig. S8f). The translation of *Gpx4* mRNA was then examined, and it was enhanced by YTHDC2 overexpression, while suppressed by *Ythdc2* knockdown (*P* < 0.01, Fig. 6c). DHX58-promoted GPX4 protein levels were also dependent on YTHDC2 (Fig. 6d). Thus, DHX58 cooperatively enhances *Gpx4* mRNA translation by recruiting YTHDC2.

Since YTHDC2 is a typical m^6^A reader that enhances the translation of m^6^A-modified mRNAs [26], we examined whether *Gpx4* mRNA was m^6^A-modified and read by YTHDC2. Using m^6^A immunoprecipitation and sequencing (MeRIP-seq), the m^6^A peaks present in *Gpx4* transcript were determined and the sequence motifs modified at the top were identified (Fig. 6e). The m^6^A modification of *Gpx4* mRNA (*P* < 0.01) was also confirmed using MeRIP-qRT-PCR (Fig. 6f). To investigate whether m^6^A-modified *Gpx4* mRNA promotes its translation, we knocked down the m^6^A writer *Mettl3* to suppress m^6^A modification (*P* < 0.01, Additional file 1: Fig. S8g), and *Gpx4* protein levels decreased (Fig. 6g), whereas its mRNA levels remained unchanged (Additional file 1: Fig. S8h), which was similar to repression observed with DHX58 or YTHDC2. Furthermore, the overexpression of DHX58 or YTHDC2 increased GPX4 protein levels in an m^6^A-dependent manner, and this promotion was abolished when *Mettl3* was knocked down (Fig. 6h). Additionally, m^6^A modification of *Gpx4* mRNA was not influenced by DHX58 (Additional file 1: Fig. S8i), and its association with DHX58 was not influenced by the m^6^A modification (Additional file 1: Fig. S8j). Collectively, our findings suggest that DHX58 exerts a protective role against ferroptosis by recruiting YTHDC2 to read and promote m^6^A-dependent translation of *Gpx4* mRNA, thus enhancing GPX4 protein levels.

### IFN-α treatment stimulates DHX58 to prevent hepatic ferroptosis

**Fig. 7:**
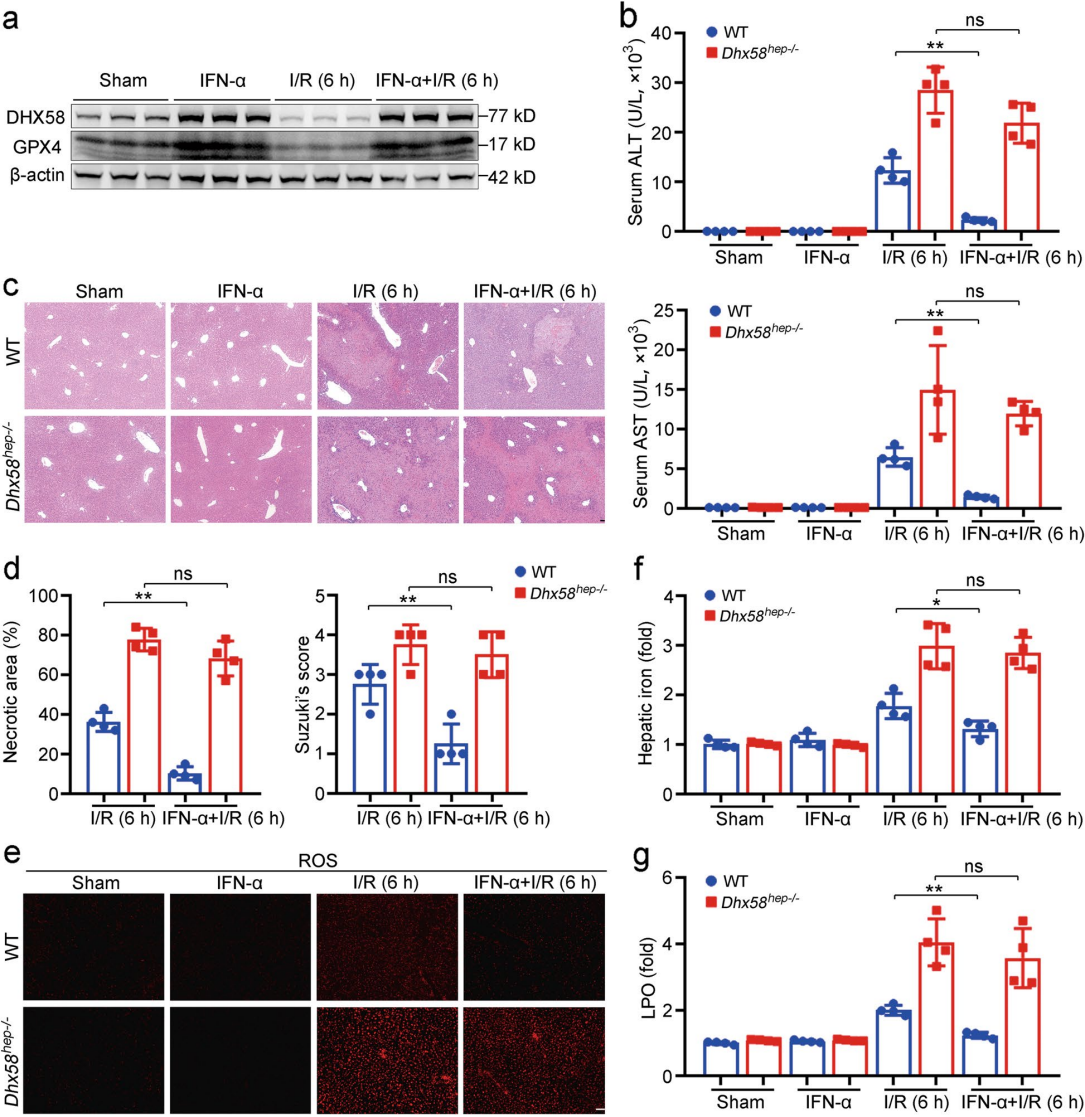
Pretreatment with IFN-α can inhibit hepatic ferroptosis by stimulating DHX58. **a** DHX58 and GPX4 protein levels in the liver with IFN-α pretreatment and then I/R. Wild-type (WT) and *Dhx58*^*hep/−*^ mice were pretreated with IFN-α, and then underwent I/R, liver damage was examined by serum ALT and AST (**b**), liver pathology was analyzed by HE staining (**c**), necrotic area and Suzuki’s score were examined (**d**), ROS production was analyzed by DHE staining (**e**), hepatic iron (**f**) and LPO (**g**) levels were analyzed accordingly. Scale bar = 20 μm. Data are shown as mean ± SD (*n* = 4) or photographs from one representative of three independent experiments. ^*^P < 0.05, ^**^P < 0.01. ns non-significant, IFN-α interferon-α, DHX58 DExH-box helicase 58, GPX4 glutathione peroxidase 4, I/R ischemia/reperfusion, ALT alanine aminotransferase, AST aspartate aminotransferase, HE hematoxylin-eosin, ROS reactive oxygen species, DHE dihydroethidium, LPO lipid peroxide, SD standard deviation

**Fig. 8:**
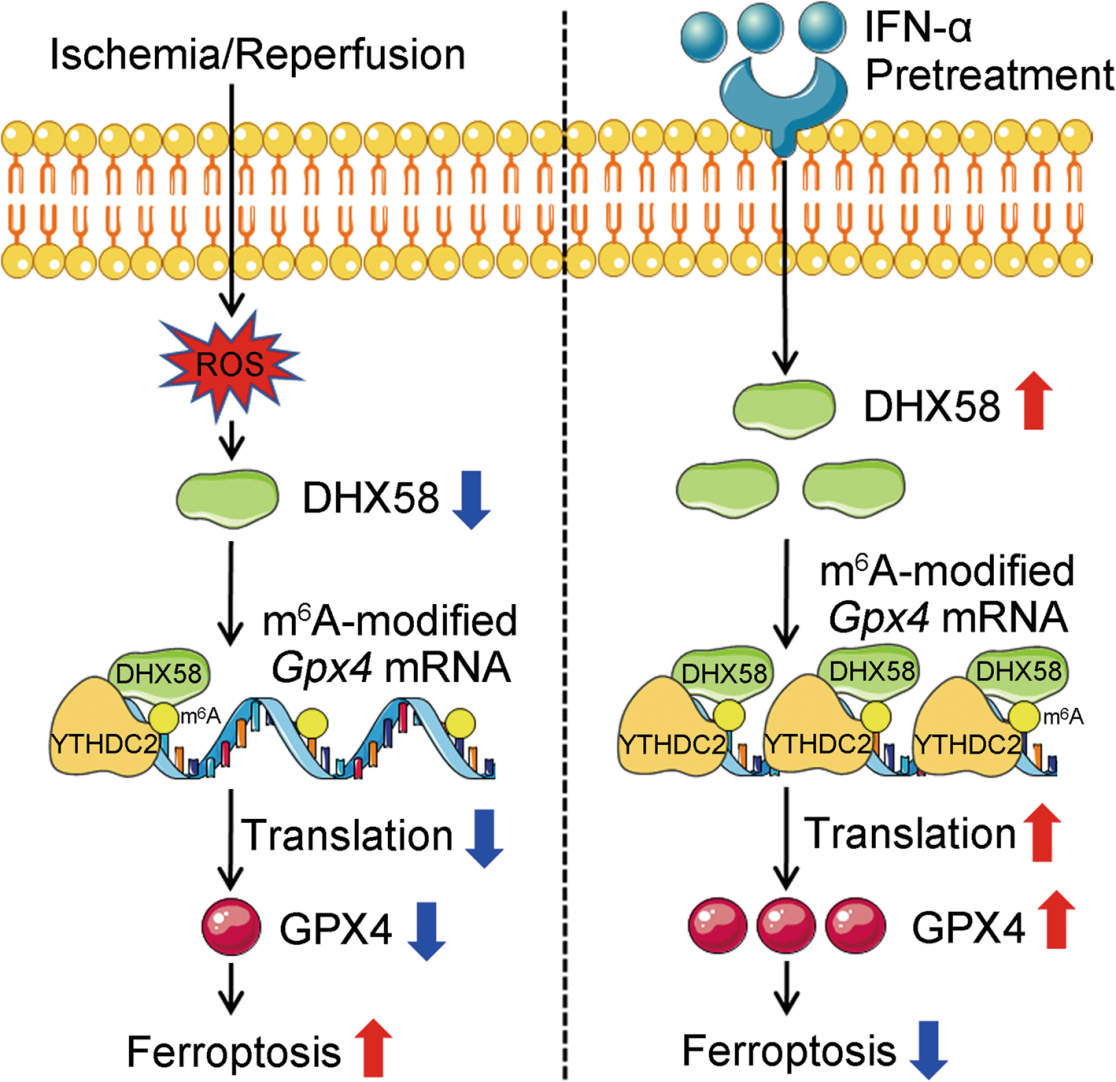
The pretreatment with IFN-α induces the activation of DHX58, which recruits YTHDC2 to recognize and enhance the translation of m^6^A-modified *Gpx4* mRNA, thus preventing hepatic ferroptosis. IFN-α interferon-α, DHX58 DExH-box helicase 58, GPX4 glutathione peroxidase 4, ROS reactive oxygen species, YTHDC2 YT521-B homology domain containing 2, m^6^A N^6^-methyladenosine

As a typical ISG, DHX58, is upregulated by IFN-α treatment [37]. We hypothesize that the induction of DHX58 expression by IFN-α treatment may potentially prevent ferroptosis during liver I/R injury. Intraperitoneal injection of IFN-α 12 h before surgery significantly promoted the expression of DHX58 and its downstream GPX4 (Fig. 7a), and liver damage was suppressed (*P* < 0.01, Fig. 7b-d), especially hepatic ferroptosis was prevented (*P* < 0.05, Fig. 7e-g; Additional file 1: Fig. S9a, b), accompanied by ameliorated expression of transferrin, hepcidin, and ferroportin after liver I/R injury (Additional file 1: Fig. S9c**)**. Moreover, the preventive effect of IFN-α was abolished in *Dhx58*^*hep−/−*^ mice (Fig. 7b-g; Additional file 1: Fig. S9a, b), determining that IFN-α treatment stimulates DHX58 expression, thereby preventing hepatic ferroptosis. Moreover, pretreatment with IFN-α also exhibited inhibitory effects on liver damage (*P* < 0.01) and ferroptosis (*P* < 0.05) in erastin-induced hepatic ferroptosis, which was abolished by *Dhx58*^*hep−/−*^ (Additional file 1: Fig. S9d-f), thereby confirming the prevention role of IFN-α-stimulated DHX58 in ferroptosis. In conclusion, we propose that excessive production of ROS during liver I/R injury leads to a decrease in DHX58 expression, thereby inhibiting downstream GPX4 and promoting ferroptosis. However, IFN-α can stimulate the expression of DHX58, leading to the recruitment of YTHDC2 to read and promote the translation of m^6^A-modified *Gpx4* mRNA, thus preventing hepatic ferroptosis (Fig. 8).

## Discussion

Liver I/R injury is an unavoidable leading cause of surgery-related liver injury and commonly occurs during war wounds and trauma. Ferroptosis in hepatocyte plays a critical role in this process, characterized by excessive ROS production and iron-dependent lipid peroxidation [38]. Here, IFN-α pretreatment exerts a protective effect against liver I/R injury, potentially through the upregulation of DHX58 expression and promotion of m^6^A-modified *Gpx4* mRNA translation, thus attenuating ferroptosis during liver I/R injury. Since the lack of effective and safe clinical precautionary or therapeutic measures remains a significant challenge in the prevention of liver I/R injury, we suggest that the treatment of IFN-α may have considerable clinical potential for preventing hepatic ferroptosis and inhibiting liver I/R injury. However, it is important to note that these findings are based on mouse models and still need validation in human subjects.

The roles of IFN in the progression of ferroptosis have been extensively studied, and multiple reports have demonstrated that IFN-γ, specially produced by T cells, can effectively promote ferroptosis in cancer cells [39–46], which appears to contradict the preventive role of IFN-α on ferroptosis in hepatocytes observed in this study. This discrepancy may be due to the different functions between IFN-α and IFN-γ, as well as the disparate responses exhibited by hepatocytes and cancer cells towards the IFN system. Although DHX58 is a typical ISG downstream of IFN, its induction in response to IFN-α and IFN-γ may exhibit variations, which could also differ between hepatocytes and cancer cells. Additionally, the precise role of DHX58 in regulating ferroptosis may vary between hepatocytes and cancer cells. Thus, additional research is necessary to explore the distinctions between IFN-α and IFN-γ in their regulation of ferroptosis, including the downstream ISGs, particularly within the context of cancer. Moreover, a previous report has also mentioned that IFN-α derived from liver plasmacytoid dendritic cells (pDCs) can promote liver I/R injury by enhancing apoptosis through the promotion of IFN regulatory factor 1 (IRF-1) [47]. Regarding the potential contradiction of IFN-α in the prevention of ferroptosis in hepatocyte determined in this study, we propose that the discrepancy may be attributed to the quantity of IFN-α in the liver. It is evident that pDCs account for only a small portion of cells in the liver, and IFN-α derived from them might have a limited impact on the overall significant elevation of IFN-α levels in the liver. Consequently, the pDCs-derived IFN-α may not be adequate to comprehensively induce the expression of the IFN-stimulated gene *Dhx58* in the liver and subsequently prevent ferroptosis in hepatocyte. In our study, we administered recombinant IFN-α through intraperitoneal injection, which effectively increased its concentration systematically in the liver and significantly enhanced protein levels of hepatic DHX58 as well as downstream GPX4, which successfully prevented ferroptosis in hepatocyte after liver I/R injury. However, further investigation is required to validate these hypotheses, especially involving human participants.

IFN-α has been used clinically to treat chronic viral infectious diseases, and its safety profile has been validated. Although influenza-like symptoms such as fever are sometimes inevitable, these symptoms often vanish within 12 h post IFN-α injection. In this study, the pretreatment of IFN-α is performed 12 h before liver I/R surgery, with a single dose of approximately 2 × 10^4^ U per mouse. This dosage (converted into human about 4 × 10^6^ U) is lower than the conventional therapeutic dose used for human viral infection or cancer, which is (0.5–1.5) × 10^7^ U per day [48, 49]. Importantly, no abnormalities were observed in liver histology and serum markers associated with liver injury after IFN-α pretreatment, suggesting that the pretreatment of IFN-α is safe for preventing ferroptosis in hepatocyte during liver I/R injury. Nonetheless, further validation in human participants is necessary to evaluate the clinical efficacy and safety of IFN-α pretreatment in the prevention of ferroptosis and I/R injury during liver operation.

During liver I/R injury, damaged mitochondria generate excessive ROS, leading to oxidative stress in hepatocytes, which is one of the main causes of hepatocyte injury and cell death. The present study discovered that ROS produced during liver I/R injury suppresses the expression of DHX58, and this reduction of DHX58 was shown to participate in the progression of ferroptosis in hepatocyte following I/R injury. Both mRNA and protein levels of DHX58 were observed to decrease after liver I/R injury or exposure to ROS, indicating that elevated ROS levels hinder DHX58 expression at the transcriptional level. We investigated the upstream promoter region of the *Dhx58* gene, as well as the possible transcription factors responsible for its basal expression in hepatocytes, and their potential interaction with oxidative stress and host antioxidant response. However, no conclusive evidence was found in this regard. Perhaps the alteration of epigenetic factors contributes to the decreased DHX58 following stimulation with excessive ROS, such as modifications at the DNA or histone levels. In particular, intracellular metabolic reprogramming induced by I/R injury and oxidative stress in hepatocytes, metabolic disorders including lactic acid, acetyl coenzyme A, and crotonyl coenzyme A, have been shown to participate in the epigenetic modifications of histones and modulate downstream gene expression under cellular stress conditions [50]. Further metabolic and epigenetic studies are required to validate this hypothesis.

DHX58, also known as LGP2, belongs to the RIG-I (DDX58)-like receptor family and serves as a well-established intracellular sensor for host recognition of invading RNA viruses in innate immune cells. In the present study, DHX58 was also observed to be expressed in parenchymal cells of organs such as hepatocytes, which may imply a specific role associated with organ function. Combined with our previous findings demonstrating predominant expression of the innate sensor RIG-I (DDX58) in parenchymal hepatocytes of the liver and its involvement in regulating hepatic lipid metabolism and inflammation [20, 21], we propose that other immune sensors or immune molecules may also exhibit expression in the parenchymal cells of various organs or systems. Moreover, their biological functions might extend beyond immune activation or regulation. These sensors possess the capability to recognize and bind nucleic acids from invading pathogens, as well as host DNA or RNA [51], thereby modulating their biological processes and functions, consequently governing the corresponding physiological or pathological processes in specific organs. This assumption hypothesis holds promise for future research.

Post-transcriptional modifications of mRNAs, such as the m^6^A modification investigated in this study, play a crucial role in regulating various biological processes, including splicing, transport, stability, and translation. These modifications are mediated by a group of enzymes known as “writers”, “erasers”, and “readers” [24]. However, the specific mechanisms through which these enzymes modify or recognize their target mRNAs remain an important scientific question due to the widespread occurrence of post-transcriptional modifications in host mRNAs. RBPs possess sequence-specific or selective RNA binding activity and it is speculated that they interact with the corresponding enzymes of post-transcriptional modifications and confer mRNA specificity for their enzymatic activities and modification. In this study, DHX58, an RBP, was found to specifically bind to *Gpx4* mRNA and recruit YTHDC2 in an m^6^A-dependent manner, thereby synergistically enhancing its translation. This finding provides a representative working model supporting the aforementioned hypothesis. Nevertheless, considering that there exists a diverse range of proteins or protein families capable of binding RNAs with specificity towards RNA-binding with specific sequences or structures, it is plausible that these proteins may also associate with post-transcriptionally modified enzymes and provide these enzymes with RNA specificity. Exploring this hypothesis could lead to intriguing future research directions within the fields of RNA modification and protein-RNA interactions.

## Conclusions

In summary, this study highlights a promising strategy for preventing hepatic ferroptosis and I/R injury by inhibiting ferroptosis in hepatocytes through upregulating the protein levels of DHX58 expression and its downstream GPX4. Mechanistically, DHX58, possessing RNA-binding activity, constitutively associates with the mRNA of *Gpx4* and recruits the m^6^A reader YTHDC2 to promote the translation of *Gpx4* mRNA in an m^6^A-dependent manner, thus enhancing GPX4 protein levels and effectively preventing hepatic ferroptosis.

### Supplementary Information

**Additional file 1: Table S1 **The primers used in this study. **Fig. S1** DHX58 expression is markedly decreased post I/R in the liver. **Fig. S2** ROS decreases DHX58 expression in hepatocytes. **Fig. S3** Hepatocyte-specific DHX58 deficiency promotes I/R‑induced liver injury and inflammation. **Fig. S4** scRNA-seq analysis of *Dhx58*^f/f^ and *Dhx58*^hep‑/-^ livers post I/R. **Fig. S5** *Dhx58*^hep‑/-^ promotes ferroptosis in hepatocyte during liver I/R injury. **Fig. S6** DHX58 inhibits ferroptosis in hepatocyte during liver I/R injury **Fig. S7** DHX58 enhances GPX4 protein level to suppress ferroptosis. **Fig. S8** DHX58 associates YTHDC2 to read and promote the translation of* Gpx4 *mRNA in an m^6^A-dependent manner. **Fig. S9** Pretreatment with IFN-α can inhibit hepatic ferroptosis by stimulating DHX58.

## Data Availability

The materials of this study are available from the corresponding author (Jin Hou) upon reasonable request and through collaborative investigations. The accession numbers of the RNA-seq data: PRJNA807799, scRNA-seq data: GSE231934, MeRIP-seq data: GSE231842, RIP-seq data: GSE231843, and 4D label free proteomics analysis data in ProteomeXchange dataset: PXD042083. All the unprocessed gels and images, and the original source data for all figures are available at Mendeley Data Reserved https://data.mendeley.com/datasets/g2tk2262sz/1.

## Abbreviations

ACSL4: Acyl-CoA synthetase long-chain family member 4
ALKBH5: AlkB homolog 5
ALT: Alanine aminotransferase
AST: Aspartate aminotransferase
ActD: Actinomycin D
BCA: Bicinchoninic acid
BHA: Butylated hydroxyanisole
CARD: Caspase-recruiting domain
CQ: Chloroquine
Co-IP: Co-immunoprecipitation
COX2: Cyclooxygenase-2
CTD: C-terminal domain
DAPI: 4’,6-diamidino-2-phenylindole
DDX/DHX: DEAD/DExH-box helicase
DHE: Dihydroethidium
DHX58: DExH-box helicase 58
FTO: Fat mass and obesity associated gene
GPX4: GSH peroxidase 4
GSH: Glutathione
GSSG: Oxidized GSH
HE: Hematoxylin and eosin
H/R: Hypoxia/re-oxygenation
4-HNE: 4-hydroxynonenal
IFN: Interferon
IL-1β: Interleukin 1β
IL-6: Interleukin 6
IP: Immunoprecipitation
IP-MS: IP-mass spectrometry
I/R: Ischemia/reperfusion
ISG: IFN-stimulated gene
KEGG: Kyoto encyclopedia of genes and genomes
LGP2: Laboratory of genetics and physiology 2
LPO: Lipid peroxide
Ly6G: Lymphocyte antigen 6 complex locus G
MCP1: Monocyte chemoattractant protein 1
MDA: Malondialdehyde
MeRIP-seq: m^6^A immunoprecipitation and sequencing
METTL3: Methyltransferase-like 3
m^6^A: N^6^-methyladenosine
NAC: N-acetylcysteine
NPC: Non-parenchymal cell
Nec-1: Necrostatin-1
OD: Optical density
pDCs: Plasmacytoid dendritic cells
qRT-PCR: Quantitative reverse-transcription polymerase chain reaction
QuSAGE: Quantitative set analysis of gene expression
RBP: RNA-binding protein
RD: Repressor domain
RIG-I: Retinoic acid-inducible gene-I
RIP-seq: RNA immunoprecipitation-sequencing
RIP-qRT-PCR: RNA immunoprecipitation-qRT-PCR
ROS: Reactive oxygen species
scRNA-seq: Single-cell RNA sequencing
SD: Standard deviation
SLC7A11: Solute carrier family 7 member 11
UAMP: Uniform manifold approximation and projection
YTHDF1: YTH N^6^-methyladenosine RNA binding protein F1
YTHDC2: YT521-B homology domain containing 2
