# Supplementary material for: Interferon-α stimulates DExH-box helicase 58 to prevent hepatocyte ferroptosis
Source: Mil Med Res. 2024 Apr 15;11:22. doi: 10.1186/s40779-024-00524-9 (PMC11017495; doi:10.1186/s40779-024-00524-9)
Supplement: Supplementary file 1 — Additional file 1: Table S1 The primers used in this study. Fig. S1 DHX58 expression is markedly decreased post I/R in the liver. Fig. S2 ROS decreases DHX58 expression in hepatocytes. Fig. S3 Hepatocyte-specific DHX58 deficiency promotes I/R‑induced liver injury and inflammation. Fig. S4 scRNA-seq analysis of Dhx58f/f and Dhx58hep‑/- livers post I/R. Fig. S5 Dhx58hep‑/- promotes ferroptosis in hepatocyte during liver I/R injury. Fig. S6 DHX58 inhibits ferroptosis in hepatocyte during liver I/R injury Fig. S7 DHX58 enhances GPX4 protein level to suppress ferroptosis. Fig. S8 DHX58 associates YTHDC2 to read and promote the translation of Gpx4 mRNA in an m6A-dependent manner. Fig. S9 Pretreatment with IFN-α can inhibit hepatic ferroptosis by stimulating DHX58. [file 40779_2024_524_MOESM1_ESM.pdf]

Materials and methods

Cell lines and transfection

The human hepatocyte cell line HHL5 was purchased from the Type Culture Collection of the Chinese Academy of Sciences (Shanghai, China), and cultured as previously described [1]. We used JetPRIME (114-15, Polyplus-transfection, France) for the transfection of plasmids and INTERFERin (409-01, Polyplus-transfection, France) for the transfection of RNAs into mouse primary hepatocytes according to the protocol of the manufacturer [2].

Flow cytometry

Liver NPCs were isolated from the left lateral/median lobes of mouse livers after I/R and incubated with Bv605-conjugated anti-CD45, Percp.cy5.5-conjugated anti-CD11b, PE-conjugated anti-F4/80, and FITC-conjugated anti-Ly6G antibodies. Neutrophils were defined as $CD11b^{+}Ly6G^{+}$ and macrophages were defined as $CD11b^{+}F4/80^{+}$. Data were obtained using Fortessa flow cytometry and analyzed using FlowJo software (BD Biosciences, CA, USA).

Hypoxia/re-oxygenation (H/R)

The hepatocytes were seeded and cultured overnight. The medium was then replaced with serum-free DMEM. Hepatocytes were then incubated in a hypoxic condition controller (BINDER, Germany) under continuous gas flow with 1% O_2, 5% CO_2, and 94% N_2. After incubation for 2 h, the cells were maintained under normoxic conditions with 95% air and 5% CO_2 for 6 h and collected for further analysis [3].

Cell viability

Cell viability was evaluated using the methyl thiazolyl tetrazolium (MTT) assay as previously described [4]. Briefly, 1×10^5 hepatocytes were seeded into each well of a 96-well plate and the corresponding treatments were performed. After removing the media, 100 µl fresh medium containing 0.5 mg/ml MTT was added into each well and incubated at 37 °C for 4 h. The medium was then replaced by 100 µl DMSO (Sigma, USA) and plates were shaken at room temperature for 10 min. Optical density (OD) was measured at 570 nm.

Molecular cloning of genes

The related genes and their corresponding truncates mentioned in this study were amplified by PCR from cDNA obtained from mouse liver tissues, which were subsequently cloned into pcDNA3.1 vectors with Flag or V5-tag. The accuracy of the constructs was confirmed by sequencing.

MS analysis

Flag-DHX58 and its associated proteins were immunoprecipitated from hepatocytes transfected with an empty vector or Flag-DHX58. After Coomassie Blue staining, the DHX58 band and the bands with more intense signals at the Flag-DHX58 Lane were cut and then analyzed by reverse-phase nanospray liquid chromatography-tandem MS. MS analysis was performed by PTM BIO (Hangzhou, China) as previously described [1].

RNA-seq

Total RNA was isolated using the TRIzol reagent (Invitrogen, Carlsbad, CA, USA). As described previously, a cDNA library was constructed and subjected to high throughput sequencing [5]. The mRNA levels of DDX/DHX family members were analyzed.

RNA extraction, real-time quantitative RT-PCR (qRT-PCR), and mouse identification

Total RNA was isolated from liver tissues and primary hepatocytes using the TRIzol reagent according to the instructions of the manufacturer. qRT-PCR was performed using the SYBR RT-PCR kit (RR430B, TaKaRa, Dalian, China) and a LightCycler (Roche, Switzerland) as described previously [4]. The relative expression of the individual genes was normalized to that of the internal control using the $2^{-\Delta\Delta Ct}$ cycle threshold method in each sample. The primer sequences for real-time PCR and mouse identification were included in **Additional file 1: Table S1**.

Histology, immunohistochemistry, and immunofluorescence

Paraffin-embedded tissue sections were stained with hematoxylin and eosin (HE) to visualize liver pathology and calculate the necrotic area using Image Pro Plus software (v6.0) as previously described [6].

A pathologist blinded to the experimental groups scored the severity of liver I/R injury according to the score criteria of Suzuki, using a semi-quantitative grading scale of 0 – 4; 0, no liver necrosis; 1, single cell necrosis; 2, up to 30% lobular necrosis; 3, up to 60% lobular necrosis; and 4, more than 60% lobular necrosis [7]. The liver sections were stained with primary antibodies against F4/80, Ly6G, 4-HNE, or MDA for immunohistochemical staining. For immunofluorescence, TUNEL staining and ROS detection by dihydroethidium (DHE) were performed as previously described [6,8].

Liver function analysis

Liver function was determined by evaluating the levels of alanine aminotransferase (ALT) and aspartate aminotransferase (AST) in the serum using an automatic biochemical analyzer, the FDC-7000i (Shanghai, China) according to the instructions of the manufacturer.

Iron, LPO, and GSH/GSSG measurement

Iron and LPO levels, and GSH/GSSG ratio were examined using an Iron Assay Kit (A039-2, Jiancheng, Jiangsu, China), a lipid peroxidation assay kit (A106, Jiancheng, Jiangsu, China), and a total GSH/oxidized GSH assay kit (A06, Jiancheng Jiangsu, China), respectively, according to the instructions of the manufacturer.

RNA decay assay

Primary hepatocytes were isolated, seeded in 24-well plates overnight, and treated with actinomycin D (SelleckChem, USA) for the indicated times. Total RNA was extracted using TRIzol reagent and analyzed using qRT-PCR.

References

1. Zhou Y, Jia K, Wang S, Li Z, Li Y, Lu S, et al. Malignant progression of liver cancer progenitors requires lysine acetyltransferase 7-acetylated and cytoplasm-translocated G protein GαS. Hepatology. 2023;77(4):1106-21.
2. Zhou Y, Wang M, Li Y, Wang P, Zhao P, Yang Z, et al. SARS-CoV-2 Spike protein enhances ACE2

expression via facilitating Interferon effects in bronchial epithelium. Immunol Lett. 2021;237:33-41.

3. Han RH, Huang HM, Han H, Chen H, Zeng F, Xie X, et al. Propofol postconditioning ameliorates hypoxia/reoxygenation induced H9c2 cell apoptosis and autophagy via upregulating forkhead transcription factors under hyperglycemia. Mil Med Res. 2021;8(1):58.
4. Hou J, Lin L, Zhou W, Wang Z, Ding G, Dong Q, et al. Identification of miRNomes in human liver and hepatocellular carcinoma reveals miR-199a/b-3p as therapeutic target for hepatocellular carcinoma. Cancer Cell. 2011;19(2):232-43.
5. Wang L, Wen M, Cao X. Nuclear hnRNPA2B1 initiates and amplifies the innate immune response to DNA viruses. Science. 2019;365(6454):eaav0758.
6. Li Z, Zhou Y, Zhang L, Jia K, Wang S, Wang M, et al. microRNA-199a-3p inhibits hepatic apoptosis and hepatocarcinogenesis by targeting PDCD4. Oncogenesis. 2020;9(10):95.
7. Hart ML, Much C, Gorzolla IC, Schittenhelm J, Kloor D, Stahl GL, et al. Extracellular adenosine production by ecto-5'-nucleotidase protects during murine hepatic ischemic preconditioning. Gastroenterology. 2008;135(5):1739-50.e3.
8. Zeng X, Zhang YD, Ma RY, Chen YJ, Xiang XM, Hou DY, et al. Activated Drp1 regulates p62-mediated autophagic flux and aggravates inflammation in cerebral ischemia-reperfusion via the ROS-RIP1/RIP3-exosome axis. Mil Med Res. 2022;9(1):25.

Table S1 The primers used in this study

Genes	Forward (5'- 3')	Reverse (5'- 3')
qPCR primers		
mDhx58	AATTCTCGCTGATTGTGGTG	CTGTAGGATGTGATCAATGG
mMcp1	TTAAAAACCTGGATCGGAACCAA	GCATTAGCTTCAGATTTACGGGT
mIl-6	TGATGCACTTGCAGAAAACA	ACCAGAGGAAATTTTCAATAGGC
mIl-1β	GCAACTGTTCCTGAACTCAACT	ATCTTTTGGGGTCCGTCAACT
mAcsl4	CTCACCATTATATTGCTGCCTGT	TCTCTTTGCCATAGCGTTTTTCT
mSlc7a11	GGCACCGTCATCGGATCAG	CTCCACAGGCAGACCAGAAAA
mCox2	TGAGCAACTATTCCAAACCAGC	GCACGTAGTCTTCGATCACTATC
mGpx4	GCCTGGATAAGTACAGGGGTT	CATGCAGATCGACTAGCTGAG
mMettl3	CTGGGCACTTGGATTTAAGGAA	TGAGAGGTGGTGTAGCAACTT
mβ-actin	AGTGTGACGTTGACATCCGT	GCAGCTCAGTAACAGTCCGC
hDhx58	ACGGGTGTATGCGCTTCAC	TTGCGGTCATCGAACAGGG
hβ-actin	AGAAGGATTCCTATGTGGGCG	GGATAGCACAGCCTGGATAGCA
Primers for mouse identification		
Dhx58$^{f/f}$	GCAAGCCTCCTTCCTCTA	CTCGTCCACCACAATCAG
Alb-Cre wild-type	TGCAAACATCACATGCACAC	TTGGCCCCTTACCATAACTG
Alb-Cre mutant	GAAGCAGAAGCTTAGGAAGATGG	TTGGCCCCTTACCATAACTG

Dhx58 DExH-box helicase 58, *Mcp1* monocyte chemoattractant protein 1, *Il-6* interleukin-6, *Acsl4* acyl-CoA synthetase long-chain family member 4, *Slc7a11* solute carrier family 7 member 11, *Cox2* cyclooxygenase-2, *Gpx4* glutathione peroxidase 4, *Mettl3* methyltransferase complex methyltransferase-like 3

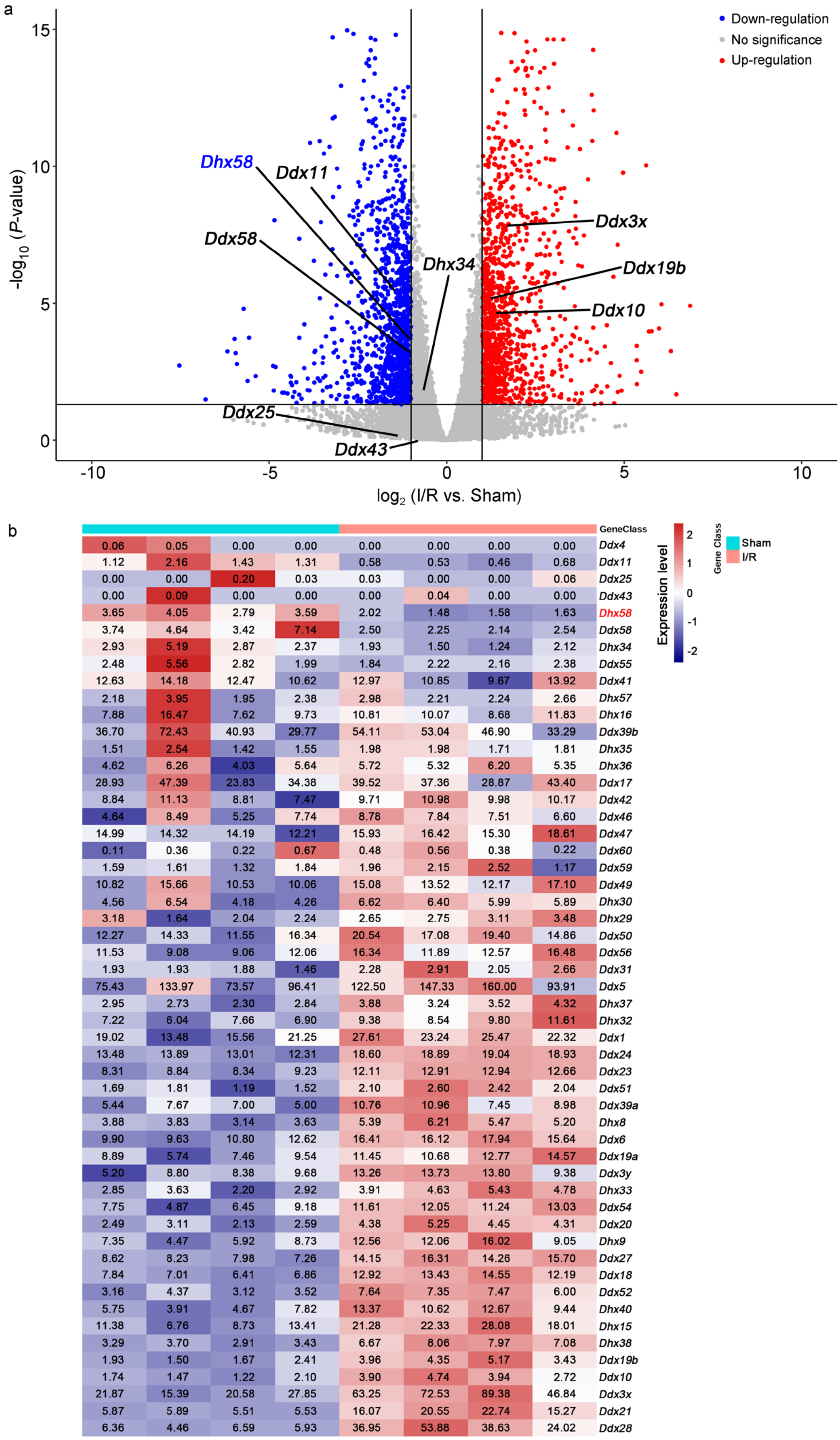

Fig. S1 DHX58 expression is markedly decreased post I/R in the liver. **a** Volcano plot of the differently expressed genes in mice liver tissues post liver I/R injury. **b** Heatmap of DDX/DHX family member expression indicated by FPKM in mice liver tissues post liver I/R injury ($n = 4$). DHX58 DExH-box helicase 58, I/R ischemia/reperfusion, DDX/DHX DEAD/DExH-box helicase, FPKM fragments per kilobase of exon

model per million mapped fragments

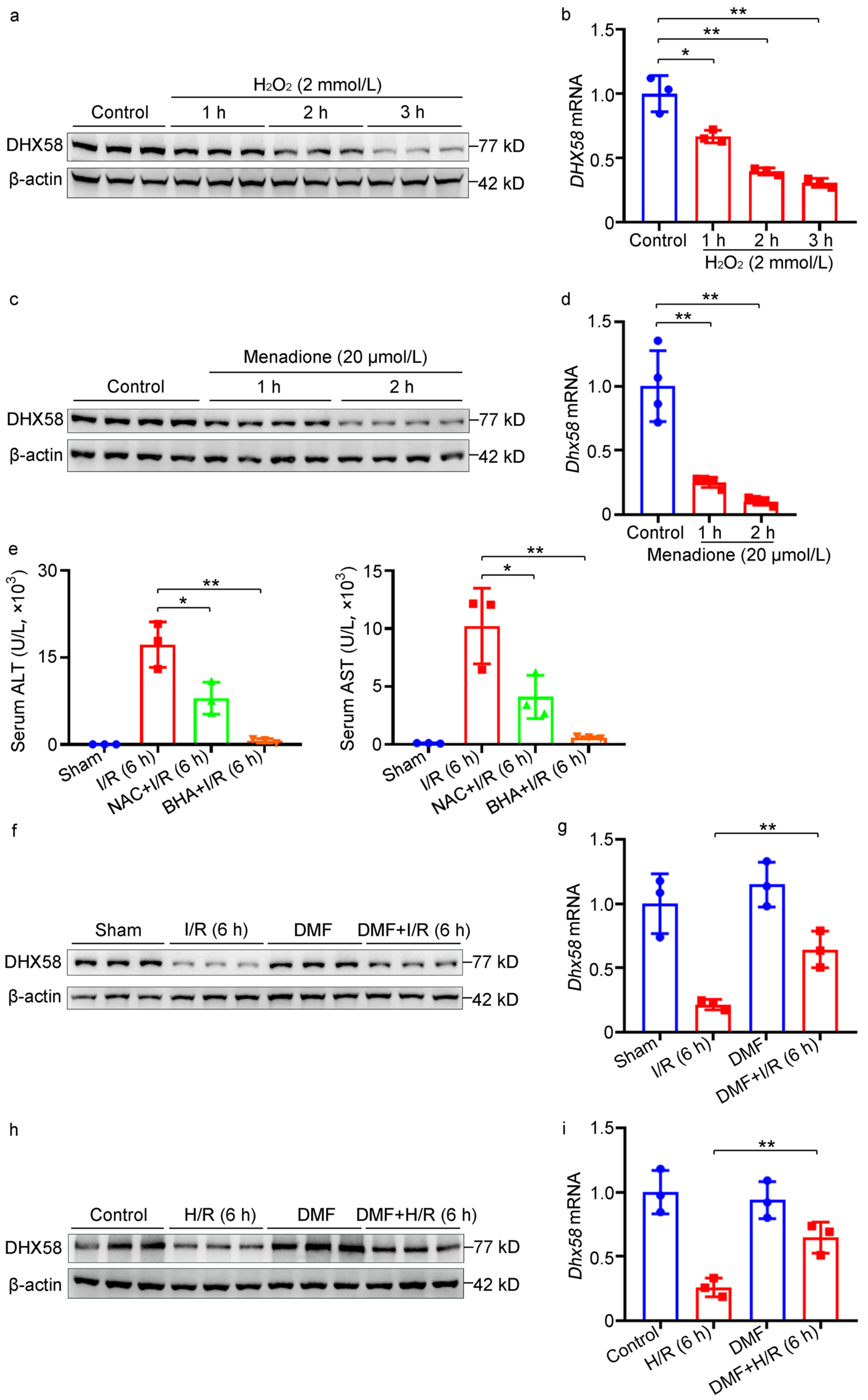

Fig. S2 ROS decreases DHX58 expression in hepatocytes. **a** DHX58 protein level in human hepatocyte cell line HHL5 treated with H_2O_2 (2 mmol/L) was examined by Western blotting. **b** *DHX58* mRNA level in human hepatocyte cell line HHL5 treated with H_2O_2 (2 mmol/L) was examined by qRT-PCR. **c** DHX58 protein level in mouse primary hepatocytes treated with menadione (20 μmol/L) was examined by Western

blotting. **d** *Dhx58* mRNA level in mouse primary hepatocytes treated with menadione (20 μmol/L) was examined by qRT-PCR. **e** Serum ALT and AST of mice with NAC or BHA pre-treatment and then I/R. DHX58 protein (**f**) and mRNA (**g**) levels in liver tissues from mice with DMF pre-treatment and then I/R were examined by Western blotting and qRT-PCR respectively. DHX58 protein (**h**) and mRNA (**i**) levels in mouse primary hepatocytes with DMF pre-treatment and then H/R were examined by Western blotting and qRT-PCR respectively. Data are shown as mean ± SD (n = 3) or photographs from one representative of three independent experiments. $^{*}P < 0.05$, $^{**}P < 0.01$. ROS reactive oxygen species, DHX58 DExH-box helicase 58, ALT alanine aminotransferase, AST aspartate aminotransferase, NAC N-acetylcysteine, BHA butylated hydroxyanisole, I/R ischemia/reperfusion, DMF dimethyl fumarate, H/R hypoxia/re-oxygenation, SD standard deviation

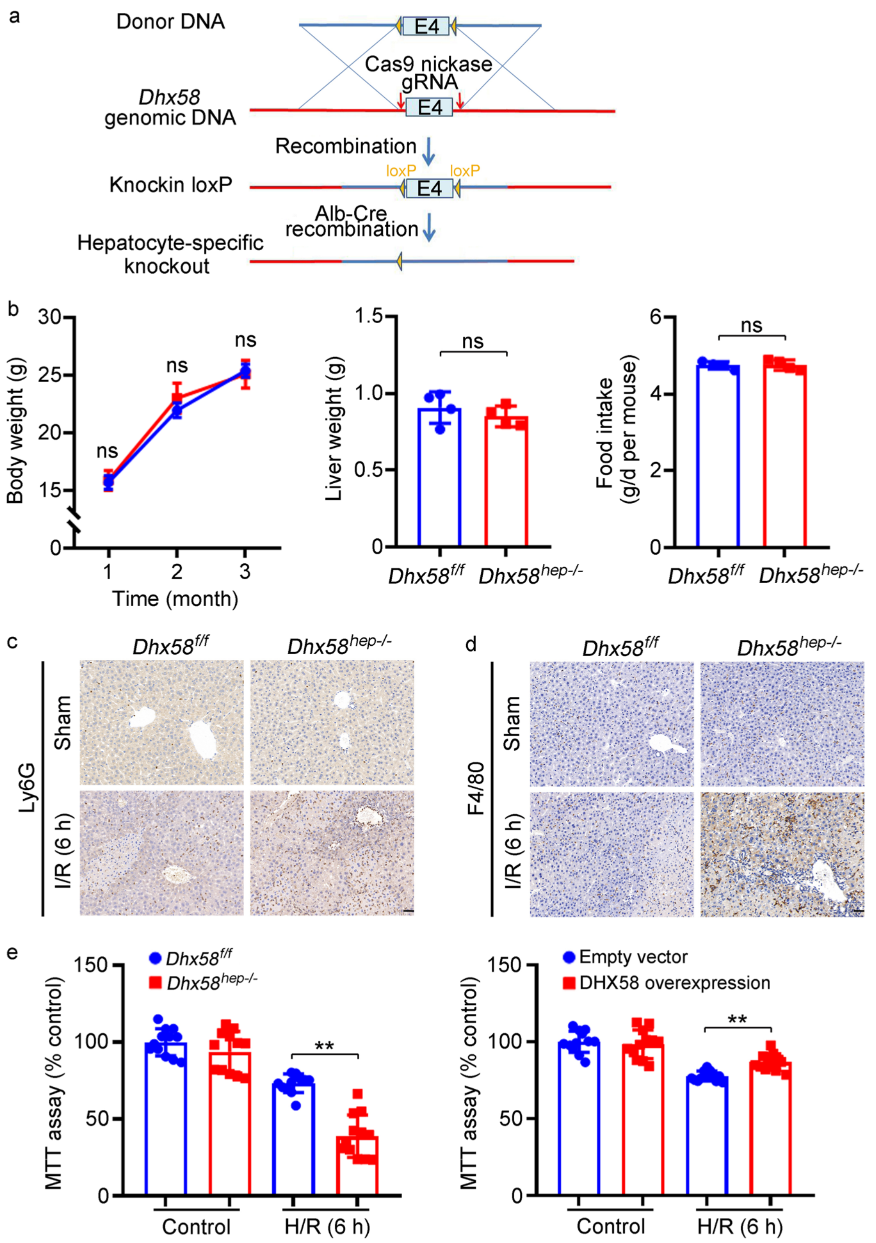

Fig. S3 Hepatocyte-specific *Dhx58* deficiency promotes I/R-induced liver injury and inflammation. **a** Construction of *Dhx58*$^{hep-/-}$ mice. **b** The body weight, liver weight, and food intake of *Dhx58*$^{f/f}$ and *Dhx58*$^{hep-/-}$ mice were shown respectively (n = 4). Liver I/R injury was administrated in *Dhx58*$^{f/f}$ and *Dhx58*$^{hep-/-}$ mice, infiltration of neutrophils (**c**) and macrophages (**d**) were analyzed by Ly6G and F4/80 staining respectively. **e** Cell viability of the primary hepatocytes with DHX58 deletion or overexpression after H/R injury was assessed by the MTT assay (n = 12). Scale bar = 20 μm. Data are shown as mean ± SD or photographs from one representative of three independent experiments. $^{*}P < 0.05$, $^{**}P < 0.01$. ns non-significant, Dhx58 DExH-box helicase 58, I/R ischemia/reperfusion, Ly6G lymphocyte antigen 6 complex locus g, H/R hypoxia/re-oxygenation, SD standard deviation, MTT methyl thiazolyl tetrazolium

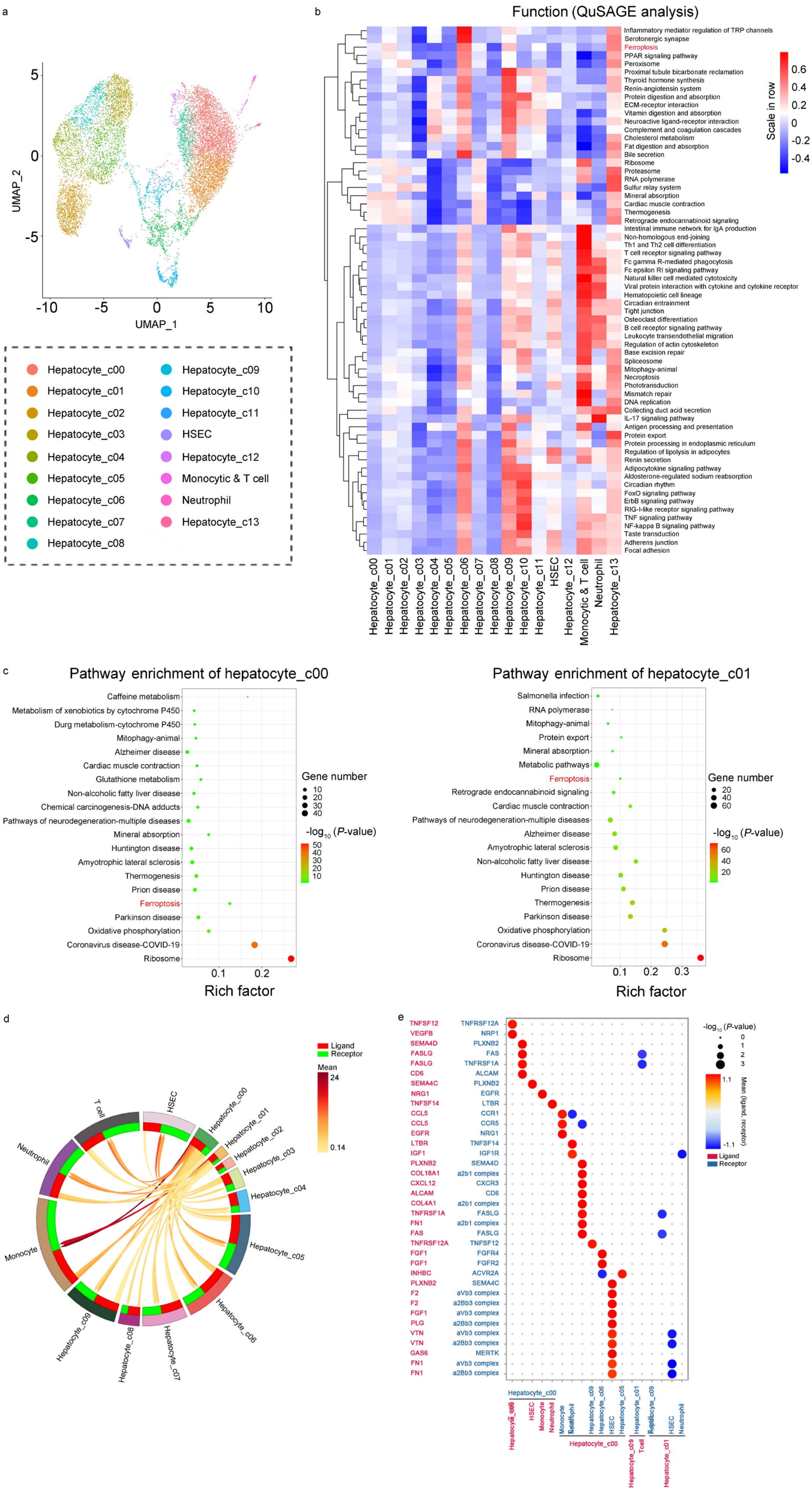
a
UMAP_1
UMAP_2
Hepatocyte_c00
Hepatocyte_c01
Hepatocyte_c02
Hepatocyte_c03
Hepatocyte_c04
Hepatocyte_c05
Hepatocyte_c06
Hepatocyte_c07
Hepatocyte_c08
Hepatocyte_c09
Hepatocyte_c10
Hepatocyte_c11
HSEC
Hepatocyte_c12
Monocytic & T cell
Neutrophil
Hepatocyte_c13
b
Function (QuSAGE analysis)
Scale in row
Inflammatory mediator regulation of TRP channels
Serotonergic synapse
Ferroptosis
PPAR signaling pathway
Peroxisome
Proximal tubule bicarbonate reclamation
Thyroid hormone synthesis
Renin-angiotensin system
Protein digestion and absorption
ECM-receptor interaction
Vitamin digestion and absorption
Neuroactive ligand-receptor interaction
Complement and coagulation cascades
Cholesterol metabolism
Fat digestion and absorption
Bile secretion
Ribosome
Proteasome
RNA polymerase
Sulfur relay system
Mineral absorption
Cardiac muscle contraction
Thermogenesis
Retrograde endocannabinoid signaling
Intestinal immune network for IgA production
Non-homologous end-joining
Th1 and Th2 cell differentiation
T cell receptor signaling pathway
Fc gamma R-mediated phagocytosis
Fc epsilon RI signaling pathway
Natural killer cell mediated cytotoxicity
Viral protein interaction with cytokine and cytokine receptor
Hematopoietic cell lineage
Circadian entrainment
Tight junction
Osteoclast differentiation
B cell receptor signaling pathway
Leukocyte transendothelial migration
Regulation of actin cytoskeleton
Base excision repair
Spliceosome
Mitophagy-animal
Necroptosis
Phototransduction
Mismatch repair
DNA replication
Collecting duct acid secretion
IL-17 signaling pathway
Antigen processing and presentation
Protein export
Protein processing in endoplasmic reticulum
Regulation of lipolysis in adipocytes
Renin secretion
Adipocytokine signaling pathway
Aldosterone-regulated sodium reabsorption
Circadian rhythm
FoxO signaling pathway
ErbB signaling pathway
RIG-I-like receptor signaling pathway
TNF signaling pathway
NF-kappa B signaling pathway
Taste transduction
Adherens junction
Focal adhesion
c
Pathway enrichment of hepatocyte_c00
Caffeine metabolism
Metabolism of xenobiotics by cytochrome P450
Durg metabolism-cytochrome P450
Mitophagy-animal
Alzheimer disease
Cardiac muscle contraction
Glutathione metabolism
Non-alcoholic fatty liver disease
Chemical carcinogenesis-DNA adducts
Pathways of neurodegeneration-multiple diseases
Mineral absorption
Huntington disease
Amyotrophic lateral sclerosis
Thermogenesis
Prion disease
Ferroptosis
Parkinson disease
Oxidative phosphorylation
Coronavirus disease-COVID-19
Ribosome
Rich factor
Gene number
-log10 (P-value)
Pathway enrichment of hepatocyte_c01
Salmonella infection
RNA polymerase
Mitophagy-animal
Protein export
Mineral absorption
Metabolic pathways
Ferroptosis
Retrograde endocannabinoid signaling
Cardiac muscle contraction
Pathways of neurodegeneration-multiple diseases
Alzheimer disease
Amyotrophic lateral sclerosis
Non-alcoholic fatty liver disease
Huntington disease
Prion disease
Thermogenesis
Parkinson disease
Oxidative phosphorylation
Coronavirus disease-COVID-19
Ribosome
Rich factor
Gene number
-log10 (P-value)
d
Ligand
Receptor
Mean
T cell
HSEC
Neutrophil
Monocyte
e
-log10 (P-value)
Mean (ligand, receptor)
Ligand
Receptor
TNFSF12 TNFRSF12A
VEGFB NRP1
SEMA4D PLXNB2
FASLG FAS
FASLG TNFRSF1A
CD6 ALCAM
SEMA4C PLXNB2
NRG1 EGFR
TNFSF14 LTBR
CCL5 CCR1
CCL5 CCR5
EGFR NRG1
LTBR TNFSF14
IGF1 IGF1R
PLXNB2 SEMA4D
COL18A1 a2b1 complex
CXCL12 CXCR3
ALCAM CD6
COL4A1 a2b1 complex
TNFRSF1A FASLG
FN1 a2b1 complex
FAS FASLG
TNFRSF12A TNFSF12
FGF1 FGFR4
FGF1 FGFR2
INHBC ACVR2A
PLXNB2 SEMA4C
F2 aVb3 complex
F2 a2Bb3 complex
FGF1 aVb3 complex
PLG a2Bb3 complex
VTN aVb3 complex
VTN a2Bb3 complex
GAS6 MERTK
FN1 aVb3 complex
FN1 a2Bb3 complex
Hepatocyte_c00

Fig. S4 scRNA-seq analysis of *Dhx58*$^{f/f}$ and *Dhx58*$^{hep-/-}$ livers post I/R. **a** UMAP visualization of cells in the livers of *Dhx58*$^{f/f}$ and *Dhx58*$^{hep-/-}$ mice post I/R. **b** Functional gene enrichments in each cell cluster of (**a**) by QuSAGE analysis are displayed. **c** Pathway enrichment analysis of the differentially expressed genes according to the KEGG database in hepatocyte_c00 and c01 clusters of **Fig. 4d** and **e**. **d** Cell communication analysis of hepatocyte_c00 and c01 clusters with other cells in the liver. **e** Potential ligand-receptor relationships of hepatocyte_c00 and c01 clusters with other cells in the liver are shown. DHX58 DExH-box helicase 58, I/R ischemia/reperfusion, UAMP uniform manifold approximation and projection, QuSAGE quantitative set analysis of gene expression, KEGG Kyoto encyclopedia of genes and genomes, HSEC hepatic sinusoidal endothelial cell, TNFSF12 tumor necrosis factor superfamily member 12, VEGFB vascular endothelial growth factor B, SEMA4D semaphorin 4D, FASLG Fas ligand, CD6 cluster of differentiation 6, SEMA4C semaphorin 4C, NRG1 neuregulin 1, TNFSF14 tumor necrosis factor superfamily member 14, CCL5 C-C motif chemokine ligand 5, EGFR epidermal growth factor receptor, LTBR lymphotoxin beta receptor, IGF1 insulin like growth factor 1, PLXNB2 plexin B2, COL18A1 collagen type XVIII alpha 1 chain, CXCL12 C-X-C motif chemokine ligand 12, ALCAM activated leukocyte cell adhesion molecule, COL4A1 collagen type IV alpha 1 chain, TNFRSF1A tumor necrosis factor receptor superfamily member 1A, FN1 fibronectin 1, FAS Fas cell surface death receptor, TNFRSF12A tumor necrosis factor receptor superfamily member 12A, FGF1 fibroblast growth factor 1, INHBC inhibin subunit beta C, PLXNB2 plexin B2, F2 coagulation factor II, thrombin, PLG plasminogen, VTN vitronectin, GAS6 growth arrest specific 6, NRP1 neuropilin 1, CCR1 C-C motif chemokine receptor 1, CCR5 C-C motif chemokine receptor 5, IGF1R insulin like growth factor 1 receptor, a2b1 complex integrin alpha 2 beta 1, CXCR3 C-X-C motif chemokine receptor 3, FGFR4 fibroblast growth factor receptor 4, FGFR2 fibroblast growth factor receptor 2, ACVR2A activin A receptor type 2A, aVb3 complex integrin alpha V beta 3 complex, a2Bb3 complex integrin alpha II B beta 3 complex, MERTK MER proto-oncogene, tyrosine kinase

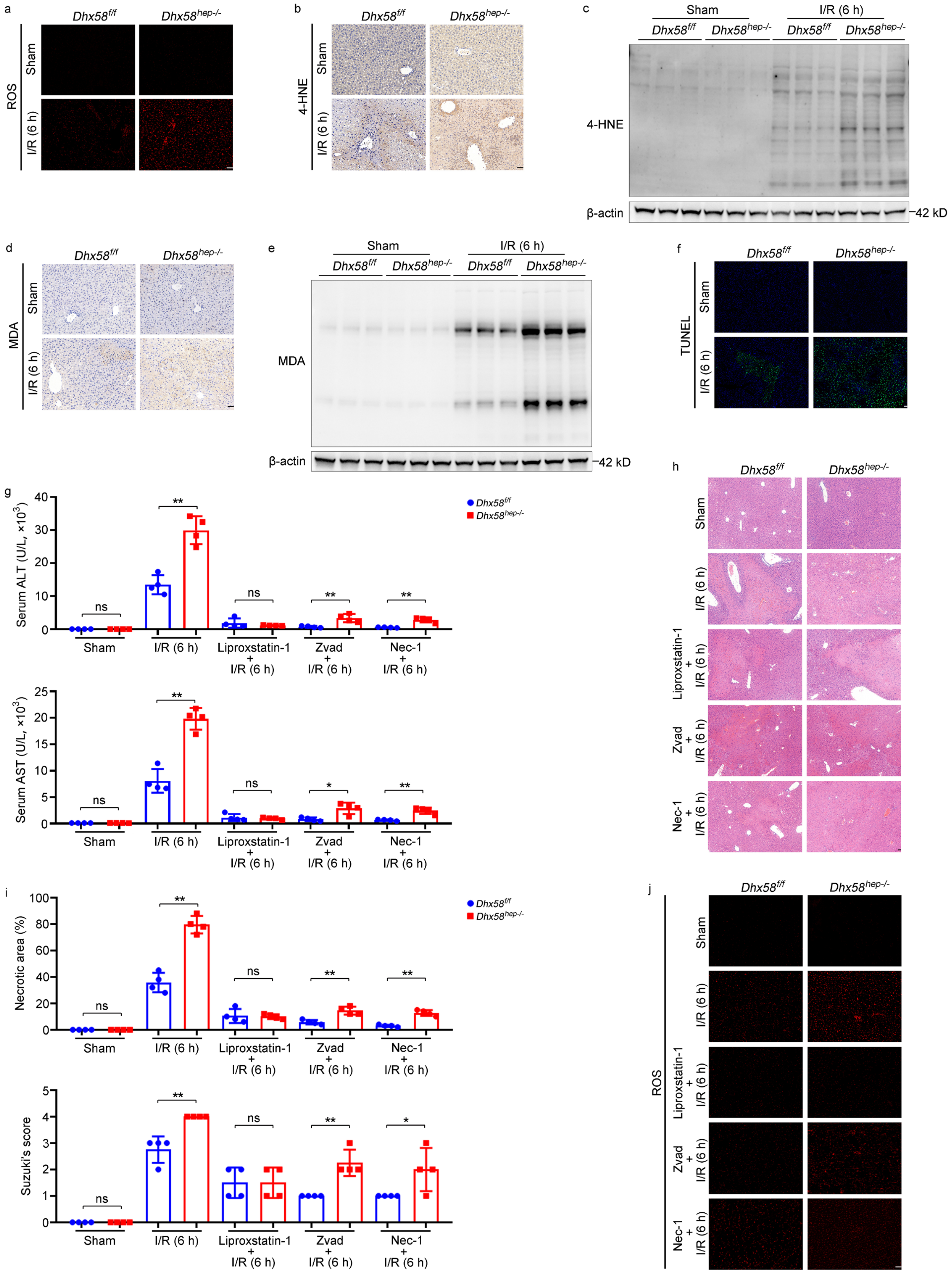

Fig. S5 *Dhx58*$^{hep-/-}$ promotes ferroptosis in hepatocyte during liver I/R injury. Liver I/R injury was administrated in *Dhx58*$^{f/f}$ and *Dhx58*$^{hep-/-}$ mice, ROS production was analyzed by DHE staining (**a**), 4-HNE was examined by immunohistochemistry staining (**b**) and Western blotting (**c**), MDA was examined by

immunohistochemistry staining (**d**) and Western blotting (**e**), TUNEL staining was performed by immunofluorescence (**f**). Liver I/R injury was administrated in $Dhx58^{f/f}$ and $Dhx58^{hep-/-}$ mice treated with liproxstatin-1, Zvad or Nec-1 respectively, serum ALT and AST were examined (n = 4) (**g**), liver pathology was examined by HE staining (**h**), necrotic area and Suzuki's score were analyzed (n = 4) (**i**), ROS production was analyzed by DHE staining (**j**). Scale bar = 20 μm. Data are shown as mean ± SD or photographs from one representative of three independent experiments. $^{*}P < 0.05$, $^{**}P < 0.01$. ns non-significant, DHX58 DExH-box helicase 58, I/R ischemia/reperfusion, 4-HNE 4-hydroxynonenal, MDA malondialdehyde, Zvad Z-VAD-FMK, Nec-1 necrostatin-1, ALT alanine aminotransferase, AST aspartate aminotransferase, HE hematoxylin-eosin, ROS reactive oxygen species, DHE dihydroethidium, SD standard deviation

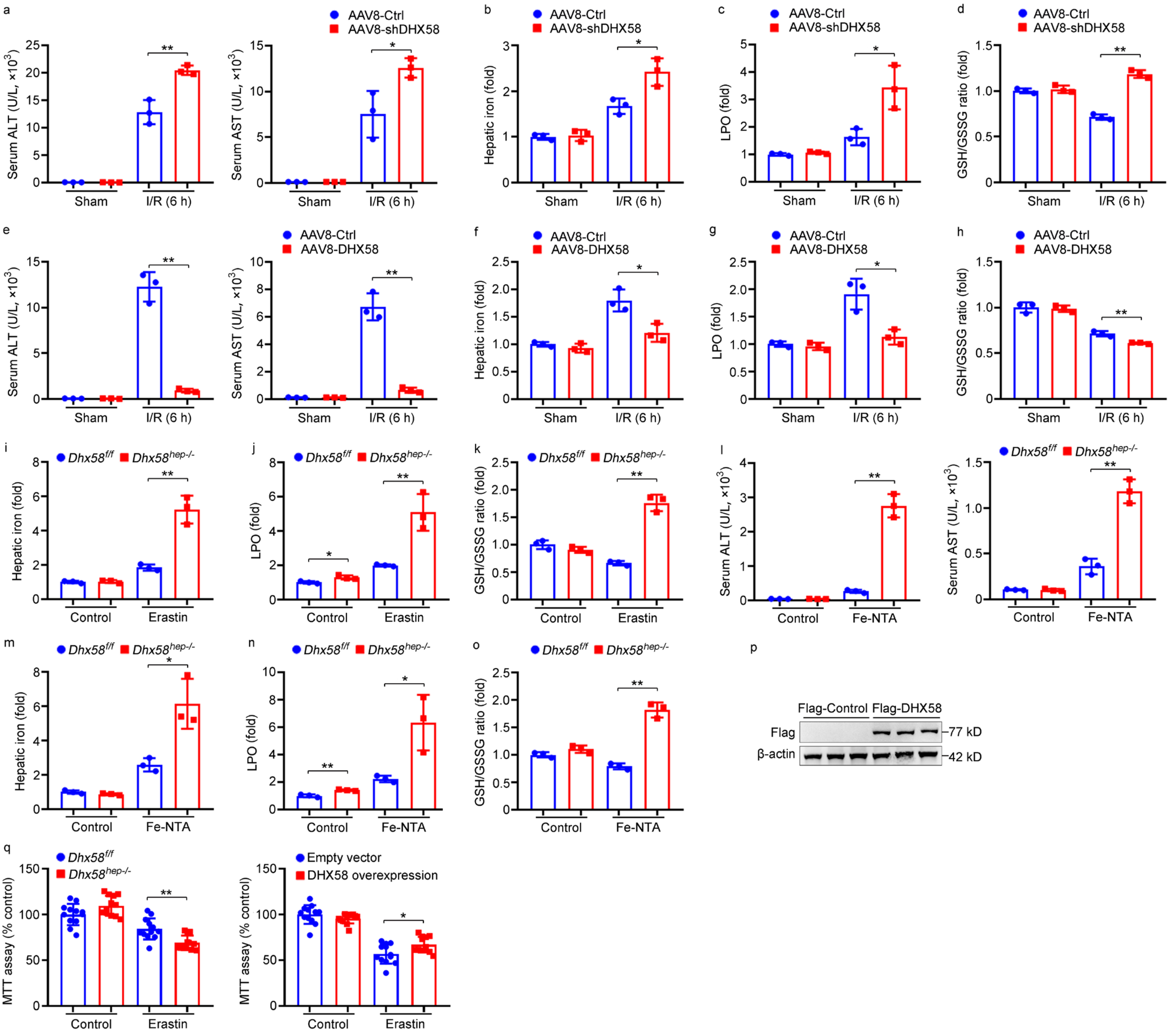

Fig. S6 DHX58 inhibits ferroptosis in hepatocyte during liver I/R injury. Liver I/R injury was administrated in mice with *Dhx58* knockdown, serum ALT and AST (**a**), hepatic iron (**b**), LPO (**c**), and the GSH/GSSG ratio (**d**) were analyzed accordingly (n = 3). Liver I/R injury was administrated in mice with DHX58 overexpression, serum ALT and AST (**e**), hepatic iron (**f**), LPO (**g**), and the GSH/GSSG ratio (**h**) were analyzed accordingly (n = 3). Hepatic iron (**i**), LPO (**j**), and the GSH/GSSG ratio (**k**) of *Dhx58*$^{f/f}$ and *Dhx58*$^{hep-/-}$ mice treated with ferroptosis inducer erastin (n = 3). Serum ALT and AST (**l**), hepatic iron (**m**), LPO (**n**), and the GSH/GSSG ratio (**o**) of *Dhx58*$^{f/f}$ and *Dhx58*$^{hep-/-}$ mice treated with ferroptosis inducer Fe-NTA (n = 3). **p** Flag-tag was examined by Western blotting in the mouse primary hepatocytes transfected with the indicated constructs. **q** Cell viability of the primary hepatocytes with DHX58 deletion or overexpression treated with ferroptosis inducer erastin was examined by MTT assay (n = 12). Data are shown as mean ± SD or photographs from one representative of three independent experiments. $^{*}P < 0.05$,

$^{**}P < 0.01$. ns non-significant, DHX58 DExH-box helicase 58, I/R ischemia/reperfusion, ALT alanine aminotransferase, AST aspartate aminotransferase, LPO lipid peroxide, GSH glutathione, GSSG oxidized glutathione, Fe-NTA ferric nitrilotriacetate, SD standard deviation

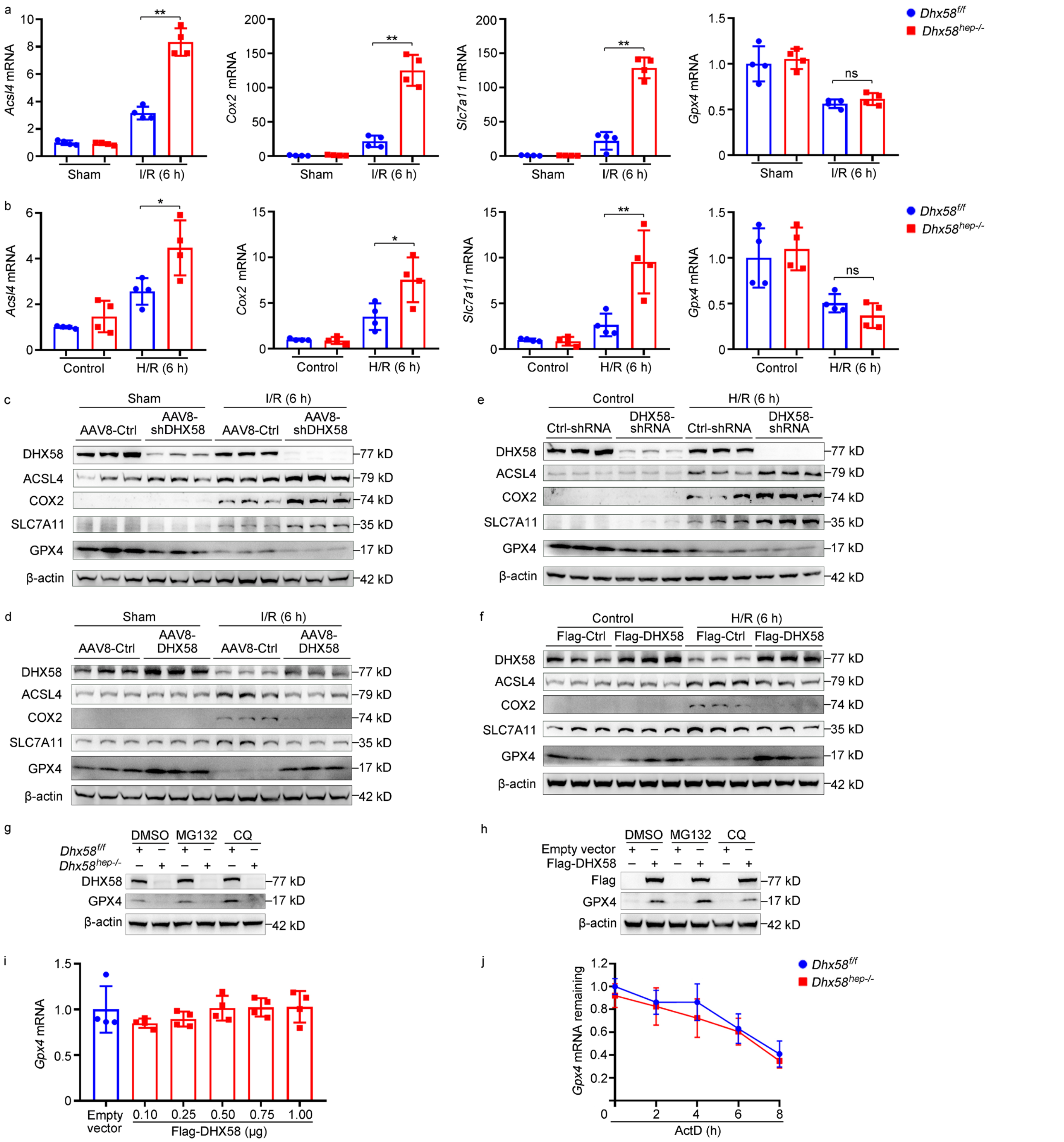

Fig. S7 DHX58 enhances GPX4 protein level to suppress ferroptosis. **a** *Acsl4*, *Cox2*, *Slc7a11* and *Gpx4* mRNA levels in liver tissues from *Dhx58*$^{f/f}$ and *Dhx58*$^{hep-/-}$ mice after liver I/R injury were examined by qRT-PCR (n = 4). **b** *Acsl4*, *Cox2*, *Slc7a11* and *Gpx4* mRNA levels in primary hepatocytes from *Dhx58*$^{f/f}$ and *Dhx58*$^{hep-/-}$ mice following H/R injury were examined by qRT-PCR (n = 4). DHX58, ACSL4, COX2, SLC7A11, and GPX4 protein levels in the livers from mice with *Dhx58* knockdown (**c**) or overexpression (**d**) and undergone I/R injury were examined by Western blotting. DHX58, ACSL4, COX2, SLC7A11, and GPX4 protein levels in the mouse primary hepatocytes with *Dhx58* knockdown (**e**) or overexpression (**f**) and

undergone H/R injury were examined by Western blotting. **g** DHX58 and GPX4 protein levels in primary hepatocytes from *Dhx58*$^{f/f}$ and *Dhx58*$^{hep-/-}$ mice treated with MG132 or CQ were examined by Western blotting. **h** Flag-tag and GPX4 protein levels in primary hepatocytes overexpressed with DHX58 and treated with MG132 or CQ were examined by Western blotting. **i** *Gpx4* mRNA level in primary hepatocytes overexpressed with DHX58 was examined by qRT-PCR (n = 4). **j** *Gpx4* mRNA level in primary hepatocytes from *Dhx58*$^{f/f}$ or *Dhx58*$^{hep-/-}$ mice treated with ActD (10 μg/ml) for the indicated time periods was examined by qRT-PCR (n = 3). Data are shown as mean ± SD or photographs from one representative of three independent experiments. $^{*}P < 0.05$, $^{**}P < 0.01$. ns non-significant, DHX58 DExH-box helicase 58, GPX4 glutathione peroxidase 4, Acsl4 acyl-CoA synthetase long chain family member 4, Cox2 cyclooxygenase-2, Slc7a11 solute carrier family 7 member 11, I/R ischemia/reperfusion, H/R hypoxia/re-oxygenation, CQ chloroquine, ActD actinomycin D, SD standard deviation

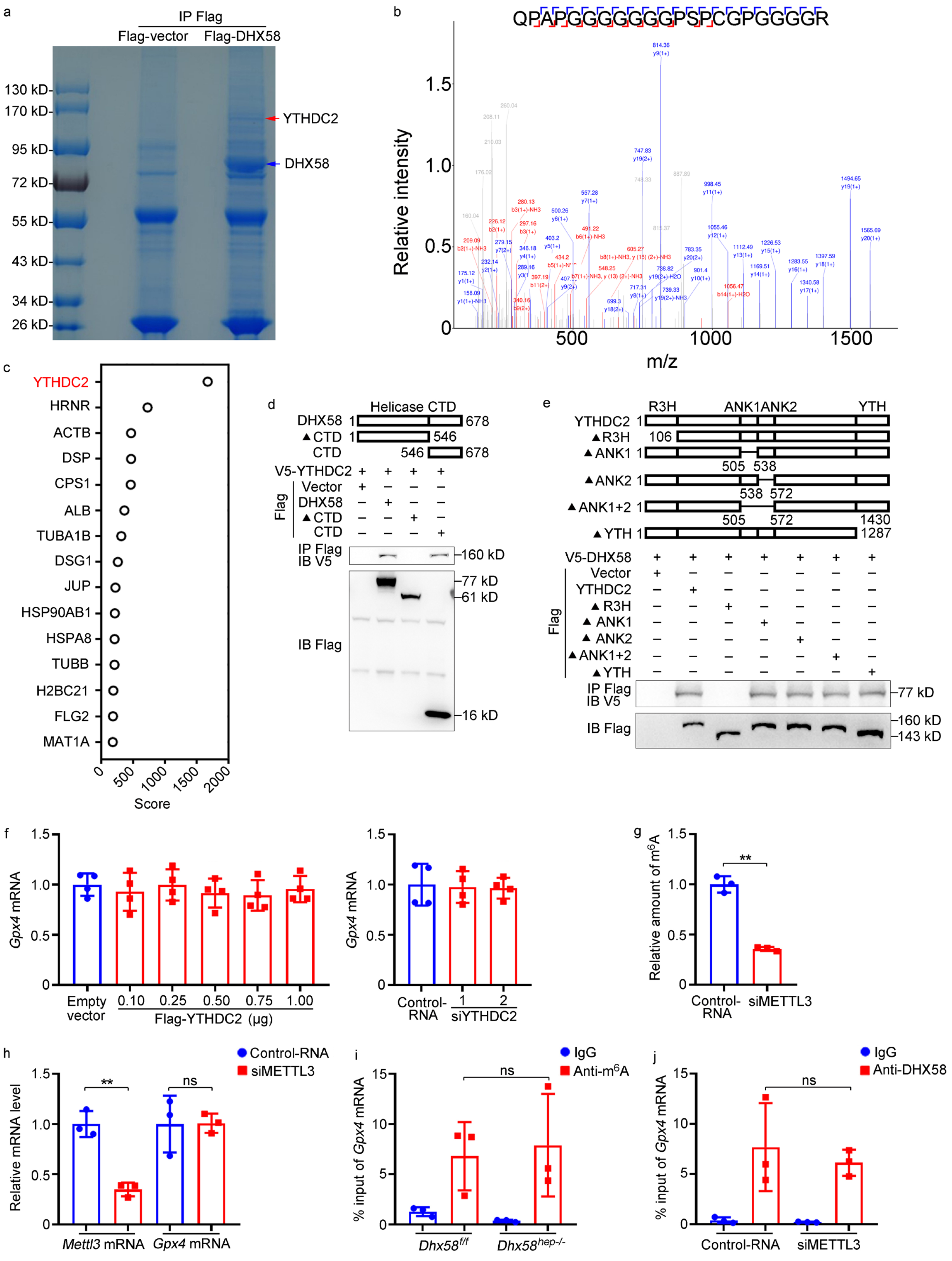

Fig. S8 DHX58 associates YTHDC2 to read and promote the translation of *Gpx4* mRNA in an m^{6}A-dependent manner. **a** PAGE gel resolution of immunoprecipitated DHX58 and its associated proteins in HHL5 cells. **b** Tandem mass spectrometry spectrum of YTHDC2 fragments. **c** Scores of DHX58-associated proteins by mass spectrometry analysis. **d** Flag-tagged DHX58 truncates were constructed, and their association with V5-tagged YTHDC2 were determined in HHL5 cells using Co-IP. **e** Flag-tagged YTHDC2

truncates were constructed, and their association with V5-tagged DHX58 were determined in HHL5 cells using Co-IP. **f** *Gpx4* mRNA level in primary hepatocytes with *Ythdc2* overexpression or knockdown was examined by qRT-PCR (n = 4). **g** Total m^{6}A modification level in primary hepatocytes with *Mettl3* knockdown was examined by m^{6}A RNA methylation assay (n = 3). **h** *Gpx4* mRNA level in primary hepatocytes with *Mettl3* knockdown was examined by qRT-PCR (n = 3). **i** m^{6}A modification of *Gpx4* mRNA in primary hepatocytes from *Dhx58*$^{f/f}$ and *Dhx58*$^{hep-/-}$ mice was examined by RIP-qRT-PCR (n = 3). **j** The association between DHX58 and *Gpx4* mRNA in primary hepatocytes with *Mettl3* knockdown was examined by RIP-qRT-PCR (n = 3). Data are shown as mean ± SD or photographs from one representative of three independent experiments or as photographs directly. $^{**}P < 0.01$. ns non-significant, ▲CTD C-terminal domain deleted, ▲R3H R3H domain deleted, ▲ANK1 ANK1 domain deleted, ▲ANK2 ANK2 domain deleted, ▲ANK1 + 2 ANK1 and 2 domains deleted, ▲YTH YTH domain deleted, DHX58 DExH-box helicase 58, YTHDC2 YT521-B homology domain containing 2, GPX4 glutathione peroxidase 4, m^{6}A N^6-methyladenosine, METTL3 methyltransferase complex methyltransferase-like 3, RIP RNA immunoprecipitation, SD standard deviation, HRNR hornerin, ACTB actin beta, DSP desmoplakin, CPS1 carbamoyl-phosphate synthase 1, ALB albumin, TUBA1B tubulin alpha 1b, DSG1 desmoglein 1, JUP junction plakoglobin, HSP90AB1 heat shock protein 90 alpha family class B member 1, HSPA8 heat shock protein family A member 8, TUBB tubulin beta class I, H2BC21 H2B clustered histone 21, FLG2 filaggrin 2, MAT1A methionine adenosyltransferase 1A, CTD C-terminal domain, ANK1 ankyrin 1, ANK2 ankyrin 2, R3H arginine-histidine-rich, YTH YT521-B homology

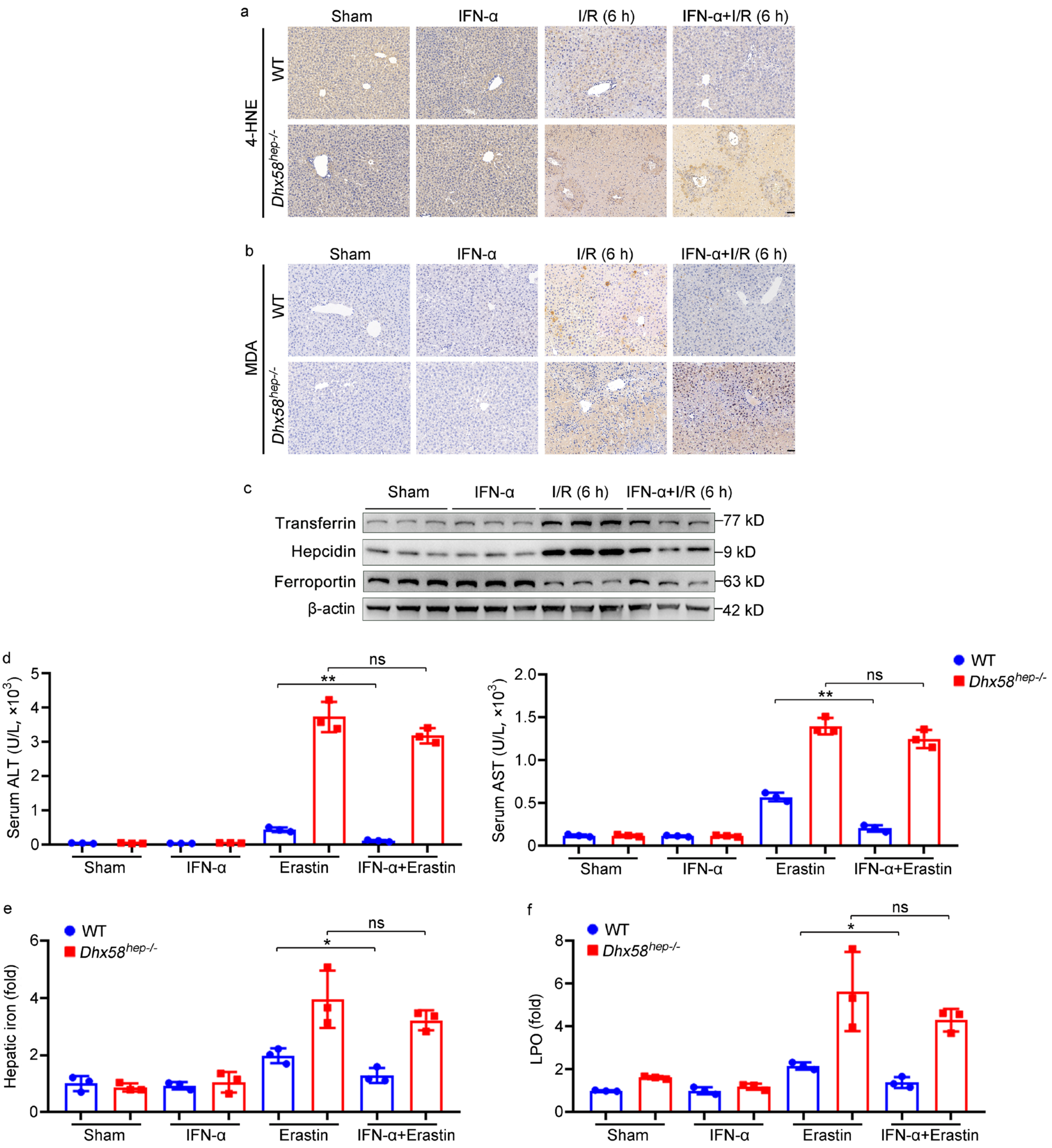

Fig. S9 Pretreatment with IFN-α can inhibit hepatic ferroptosis by stimulating DHX58. Wild-type (WT) and *Dhx58*$^{hep-/-}$ mice were pretreated with IFN-α, and then undergone liver I/R, 4-HNE (**a**) and MDA (**b**) were examined by immunohistochemistry staining. Scale bar = 20 μm. **c** Transferrin, hepcidin, and ferroportin protein levels in the livers with IFN-α pretreatment and then I/R were examined by Western blotting. WT and *Dhx58*$^{hep-/-}$ mice were pretreated with IFN-α, and then administrated with the ferroptosis inducer erastin, serum ALT and AST (**d**), hepatic iron (**e**), and LPO (**f**) levels were analyzed accordingly. Data are shown as mean ± SD (n = 3) or photographs from one representative of three independent experiments. $^{*}P < 0.05$, $^{**}P < 0.01$. ns non-significant, IFN-α interferon-α, DHX58 DExH-box helicase 58, I/R ischemia/reperfusion, 4-HNE 4-hydroxynonenal, MDA malondialdehyde, ALT alanine aminotransferase, AST aspartate

aminotransferase, LPO lipid peroxide, SD standard deviation
